# Isolation and characterization of 1-palmitoyl-2-linoleoyl-*sn*-glycerol as a hormogonium-inducing factor (HIF) from the coralloid roots of *Cycas revoluta* (Cycadaceae)

**DOI:** 10.1038/s41598-019-39647-8

**Published:** 2019-03-18

**Authors:** Yasuyuki Hashidoko, Hiroaki Nishizuka, Manato Tanaka, Kanako Murata, Yuta Murai, Makoto Hashimoto

**Affiliations:** 1 Division of Applied Bioscience, Research Faculty of Agriculture, Hokkaido University, Kita 9 Nishi 9, Kita-ku, Sapporo, 060-8589 Japan; 2 Present Address: Division of Life Science, Graduate School of Life Science, Hokkaido University, Kita 10 Nishi 8, Kita-ku Sapporo, 060-0810 Japan

## Abstract

Coralloid roots are specialized tissues of cycads (*Cycas revoluta*) that are involved in symbioses with nitrogen-fixing *Nostoc* cyanobacteria. We found that a crude methanolic extract of coralloid roots induced differentiation of the filamentous cell aggregates of *Nostoc* species into motile hormogonia. Hence, the hormogonium-inducing factor (HIF) was chased using bioassay-based isolation, and the active principle was characterized as a mixture of diacylglycerols (DAGs), mainly composed of 1-palmitoyl-2-linoleoyl*-sn*-glycerol (**1**), 1-palmitoyl-2-oleoyl*-sn*-glycerol (**2**), 1-stearoyl-2-linolenoyl*-sn*-glycerol (**3**), and 1-stearoyl-2-linoleoyl*-sn*-glycerol (**4**). Enantioselectively synthesised compound **1** showed a clear HIF activity at 1 nmol (0.6 µg) disc^−1^ for the filamentous cells, whereas synthesised 2-linoleoyl-3-palmitoyl*-sn*-glycerol (**1′**) and 1-palmitoyl-2-linoleoyl-*rac*-glycerol (**1**/**1′**) were less active than **1**. Conversely, synthesised 1-linoleoyl-2-palmitoyl-*rac*-glycerol (**8**/**8′**) which is an acyl positional isomer of compound **1** was inactive. In addition, neither 1-monoacylglycerols nor phospholipids structurally related to **1** showed HIF-like activities. As DAGs are protein kinase C (PKC) activators, 12-*O*-tetradecanoylphorbol-13-acetate (**12**), urushiol C15:3-Δ^**10**,**13**,**16**^ (**13**), and a skin irritant anacardic acid C15:1-Δ^**8**^ (**14**) were also examined for HIF-like activities toward the *Nostoc* cells. Neither **12** nor **13** showed HIF-like activities, whereas **14** showed an HIF-like activity at 1 nmol/disc. These findings appear to indicate that some DAGs act as hormogonium-inducing signal molecules for filamentous *Nostoc* cyanobacteria.

## Introduction

The cycad *Cycas revoluta* is a gymnosperm plant species that is often adapted to growth on rocky coastal cliffs under nitrogen- and phosphorus-deficient conditions. To survive in such adverse nutrient-poor conditions, *C*. *revoluta* has generally evolved a symbiotic partnership with the bacterium *Nostoc punctiforme* the cells of which are harbored within morphologically and functionally specialized symbiotic roots, referred to as coralloid roots^1^. However, *C*. *revoluta* distributed throughout South Kyushu and Ryukyu Islands allows symbiosis with not only *N*. *punctiforme* but also some other *Nostoc* species, such as *N*. *commune*^2^. Cross-sections of the thick roots have revealed that these symbiotic Nostocean cyanobacteria, which effectively fix dinitrogen gas (N_2_) into ammonia (NH_3_), are located in intercellular spaces along the endodermis: some as a short and morphologically less clear filamentous state, whereas high frequencies of the unicellular spherical heterocysts and akinetes with a larger cell size are observed (Supplementary Fig. S1)^3–6^.

Under certain environmental conditions, *Nostoc* species form motile filaments of cells referred to as hormogonia^7,8^. Irradiation with red light or weak white light has been shown to induce differentiation of filamentous cells of *Nostoc flagelliforme*, an edible non-symbiotic cyanobacterium of fat choy^9^, into hormogonia. *Calothrix*, another genus of order *Nostoceales* irradiated with red light induced differentiation to hormogonia, whereas green light rather suppressed its hormogonium induction^10,11^. The regulatory mechanisms underlying this differentiation have been investigated using mutant strains in which the green/red-perceiving histidine-kinase gene (*ccaS*) or the cognate response regulator gene (*ccaR*) were disrupted^12^. However, root-symbiotic Nostocean cyanobacteria in the soil or near the ground surface covered with topsoil often encounter weak light only.

In addition, the nutritional signals that induce hormogonium differentiation, particularly those associated with the nitrogen starvation of host plants, have also been investigated^13^. When dense filamentous colonies in the stationary phase were transferred into fresh but nitrogen-poor medium, frequent induction of hormogonium differentiation has often been observed in some Nostocean cyanobacteria, suggesting that nitrogen starvation triggers induction of hormogonia. Conversely, similar induction of hormogonia was sometimes observed even in a fresh medium containing sufficient nitrogen sources. This observation suggests the presence of hormogonia repressing factors in the spent medium, and removal of the factors should administer an indirect induction of hormogonium differentiation. However, it has for a long time been believed that hormogonium induction in symbiotic Nostocean cyanobacteria is also promoted by the host plants^8^. Given the nature of such symbiotic interactions, host cycad and Nostocean symbiont would exchange chemical signals during symbiotic events (Supplementary Fig. S1)^8,14,15^, similar to those characterizing the symbiosis between nodulation bacteria and leguminous plants^16–19^.

In this regard, host plants are known to produce exudates known as a hormogonium-inducing factors (HIFs)^14,20,21^. Hence, it has long been assumed that cycad roots produce an HIF. Indeed, a water extract of *Zamia furfuracea* seeds has been shown to induce hormogonium differentiation in *Nostoc* sp. FUR 94210, although to date, no HIFs have been identified in this extract^22^. Coralloid roots initially develop from lateral roots, the swollen root tip of which branches into two or three sections to form a pre-coralloid root at the germinated seedling stage^23,24^. *N*. *punctiforme* gains entry into the swollen root tip via a small pore in the node, and to obtain the symbionts, cycad roots need to attract the motile phase of *N*. *punctiforme* to the pre-coralloid root. Liaimer *et al*. used a polyketide synthase-disrupted mutant *pks*^2−^ for secretome analysis, and found cyclic oligopeptides, nostopeptolide A1 and A2, to be important autogenic hormogonium-repressing factors or chemoattractants toward hormogonia^25^. Thus, an HIF could be released from cycad root tips to attract Nostocean hormogonia into the symbiotic pre-coralloid root. One of the chemicals produced from the gland organ of *Gunnera* has been identified as a monosaccharide^1,3,26,27^. However, compared with the interaction between *Gunnera* and *Nostoc* cyanobacteria, biological signaling substances involved in the interaction between cycads and *Nostoc* cyanobacteria have rarely been investigated^25,28^.

We have observed that MeOH extract from coralloid roots (80 g) of cycads clearly stimulated growth of the Nostocean cyanobacterial cells in Winogradsky’s mineral solution-based nitrogen-poor medium solidified with 0.3% gellan gum^29^. To search for hypothetical nitrogen-fixation stimulating principle in the coralloid roots of *C*. *revolute*, the methanolic extract concentrated was first partitioned between water and *n*-hexane followed by EtOAc, due to partly insoluble in water, MeOH, or acetone. The EtOAc solubles fractionated by silica gel column chromatography for a stepwise elusion with 20–100% *n*-hexane-EtOAc were tested for its cell growth and acetylene reduction. In incubation at 30 °C for **5** weeks, the fraction eluted with 100% EtOAc (equivalent to 2 g of fresh coralloid roots/10 mL medium) showed pronounced cyanobacterial cell growth, suggesting the presence of certain signaling factor(s) for growth stimulation of the Nostocean cyanobacteria.

In this study, we show that 1-palmitoyl-2-linoleoyl-*sn*-glycerol and some other diacylglycerols (DAGs) show HIF-like activity toward filamentous aggregates of *Nostoc* cyanobacteria, and we further discuss the significance of hormogonium induction by DAG and other acylglycerols.

## Results

### Isolation and identification of an HIF from *C*. *revoluta* coralloid roots

**Figure 1:**
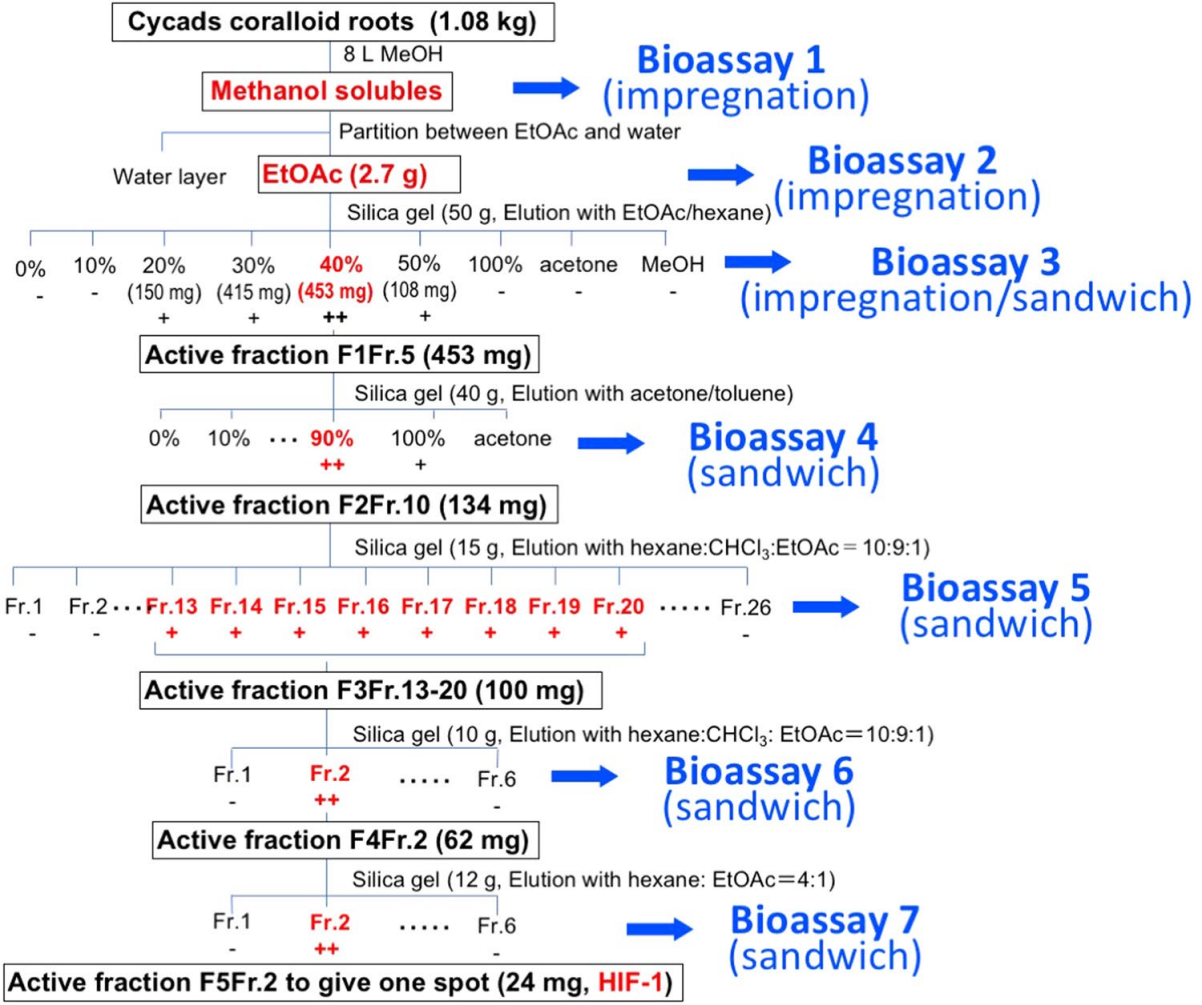
Bioassay-based isolation process for the hormogonium-inducing factor (HIF) isolated from coralloid roots of *Cycas revolute.* For each HIF-like activity, constituents in the EtOAc-soluble or the fractions equivalent to 2 g fresh weight of coralloid root are charged on paper disc. Gel for impregnation or sandwich assay are a Winogradsky’s solution-based 0.8–1.2% gellan or agar medium. After appropriate incubation of Nostocean filamentous cell colonies, cell morphology and viability are recorded using a fluorescent microscope under white light and UV light (with an excitation wave length of 580–650 nm through a Cy5 filter). During incubation from 0 to 48 h, fixed-point observations are made every 12 h from the transparent bottom-side to assess induction of Nostocean hormogonia from the filamentous cell aggregates.

After a 10-day incubation with the EtOAc-soluble preparation (equivalent to 2.0 g f.w. of the coralloid roots), the bioassay plate showed a characteristic haze-like green area around the paper disc (Supplementary Fig. S2a), whereas no such response was observed for the water-soluble preparation (1^st^ bioassay in Fig. 1). Light microscopic observations revealed that this haze-like area induced by the EtOAc-soluble preparation was filled with hormogonium-like, short and multicellular linear chains of Nostocean cells.

**Figure 2:**
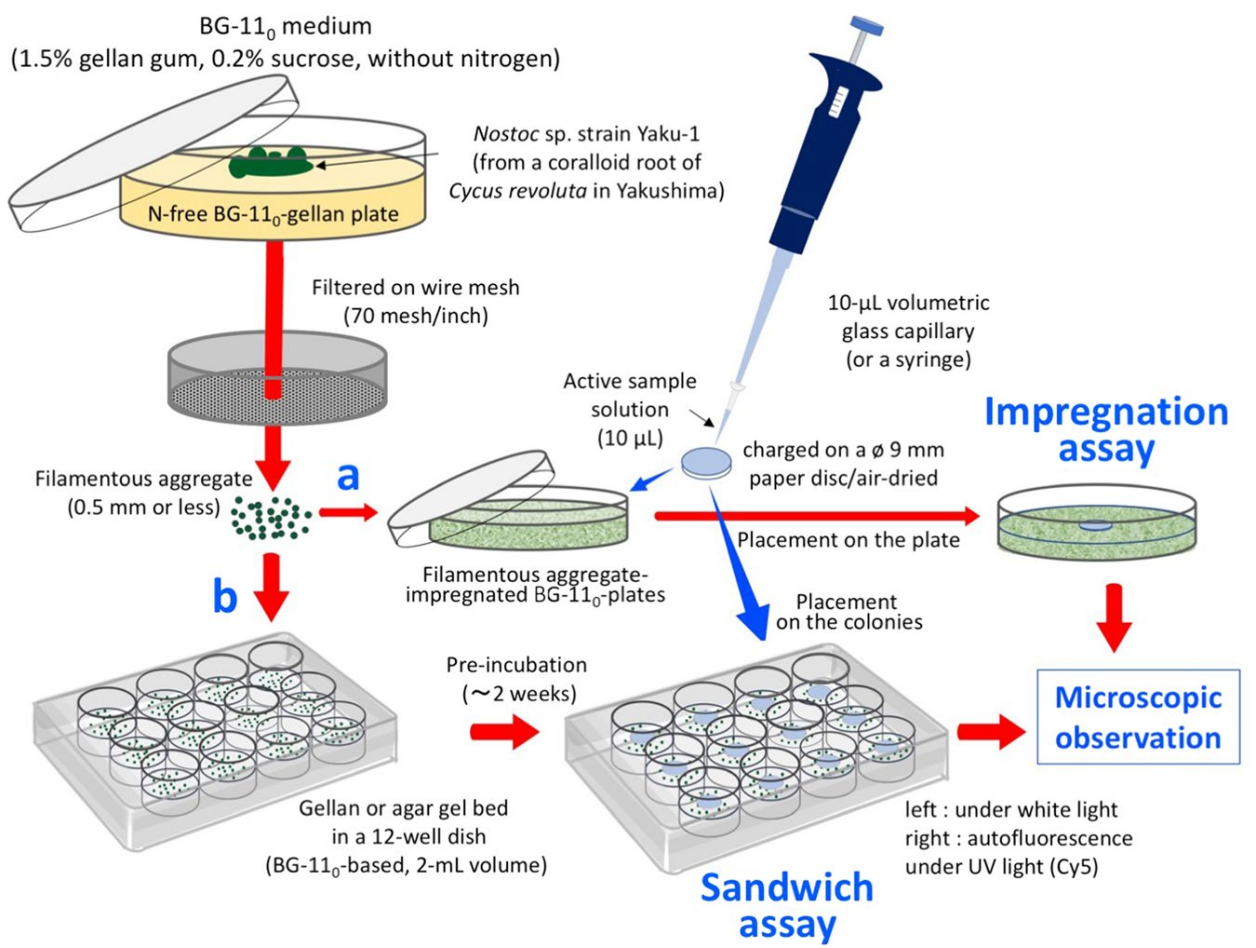
Hormogonium-inducing activity of an active fraction of an ethyl acetate-soluble preparation obtained from the coralloid roots *Cycas revolute*. (**a**) Impregnation assay. (**b**) Sandwich assay. Two bioassays in the trials are examined by the EtOAc-soluble and the extract preliminary fractionated as active Fr-5, both as those equivalent to 2 g of coralloid roots. Approximate zones shown by circles in the impregnation assay (**a**) and the area shown by squares in the sandwich assay (**b**) are magnified as its Cy5-UV or white light. Incubation time for each assay are shown in the figure.

**Figure 3:**
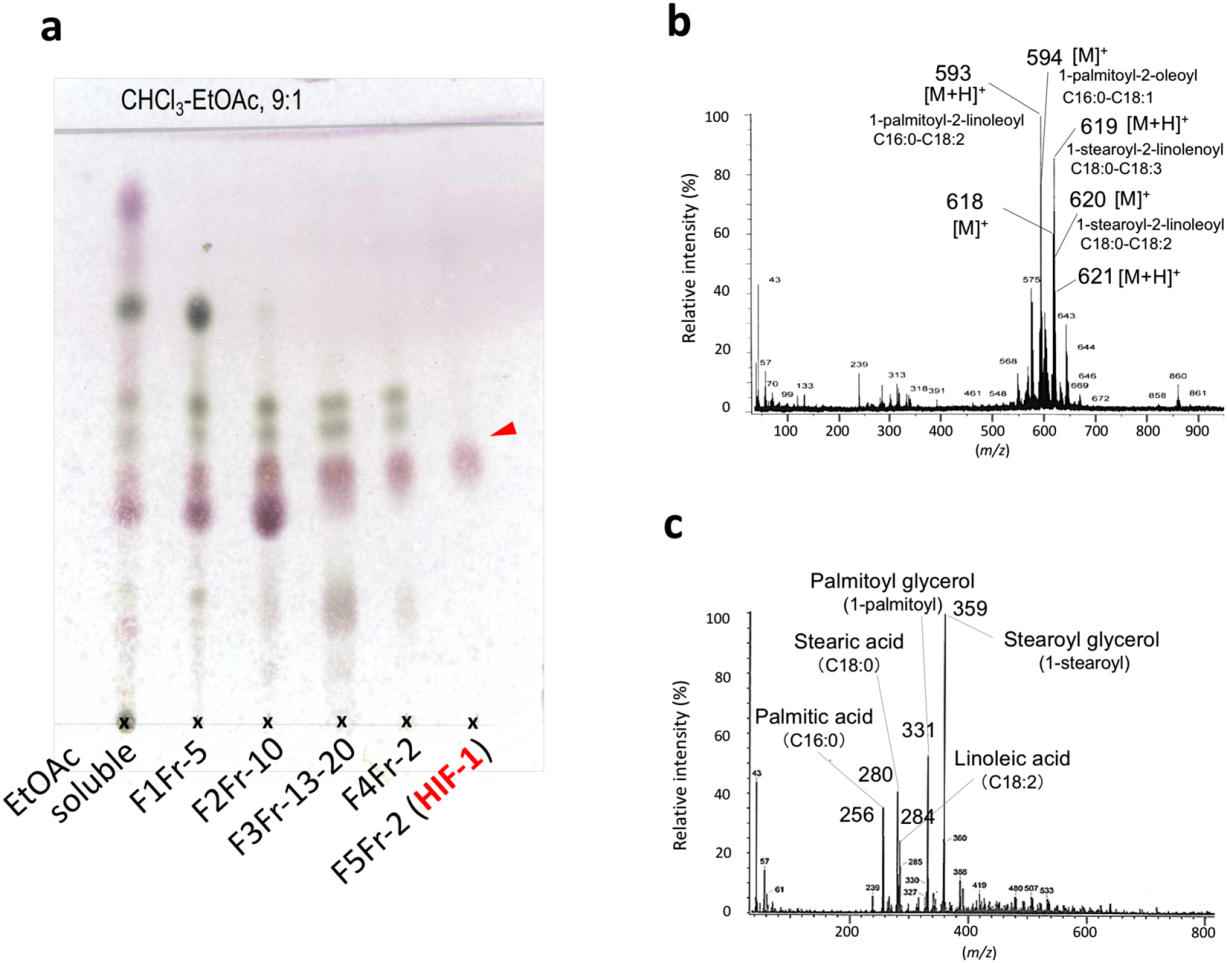
Purification of the hormogonium-inducing factor (HIF) isolated from the coralloid roots of *Cycas revoluta* and its FD-MS spectra. (**a**) Purification steps for HIF-1 from an EtOAc-soluble preparation monitored using normal-phase TLC. HIF-1 does not show any quenching spot but administers a bright pinkish colored spot by vanillin-sulfuric acid reagent. (**b**) FD-MS of the finally purified HIF-1. (**c**) FD-MS of 1-monoacylglycerol mixture obtained by a partial digestion of HIF-1 with lipase A. The mixture of monoacylglycerols obtained by a preparative TLC (hexane-EtOAc 4:1) as a spot more polar than the HIF was, and subjected to FD-MS analysis.

F1Fr-5 eluted with 40% EtOAc/hexane from the 1^st^ silica gel column chromatography (270 g of silica gel) of the EtOAc-soluble preparation (2.7 g) obtained from the methanolic extract showed clear hormogonium induction at a concentration of 91 μg disc^−1^ (equivalent to 36 mg of fresh coralloid roots) within 24 h (2^nd^ bioassay). Under a Biorevo BZ9000 fluorescent microscope (Keyence, Osaka, Japan), filamentous cell aggregates that emitted red fluorescence provided evidence that the cells were viable^30^. Motile cells differentiated from the viable and immotile Nostocean cell aggregates into short, linear chains of Nostocean hormogonia were dispersed inside solid medium in the plate. They were moving at approximately 0.7 µm s^−1^ on the gel surface, and all the motile filaments possessed two cells at both terminal ends without intrinsic fluorescence. These morphological features are characteristic of hormogonia, a motile form of Nostocean cyanobacteria (Supplementary Fig. S2b). By the bioassay-guided fractionation using sandwiched bioassay (Fig. 2), the active principle HIF-1 was obtained to administer single spot on TLC (Fig. 3a).

In the ^1^H NMR spectrum, purified HIF-1 showed a typical signal pattern identical to DAGs, in which at least one of the acyl groups is an unsaturated fatty acid (Supplementary Fig. S3). HIF-1, a mixture of DAGs, showed three main parent ions, at *m/z* 593 (100%), 618 (61%), 619 (87%), and 620 (67%) in FD-MS (Fig. 3b). Regarding these as [M + 1]^+^, the major component appearing at *m/z* 593 was estimated to be a DAG with C_16:0_-C_18:2_ acyl groups harboring two double bonds in the molecule. Similarly, *m/z* 618, and 620 were consistent with DAGs containing C_18_-C_18_ acyl groups with a total of 3 and 2 double bonds, respectively.

**Figure 4:**
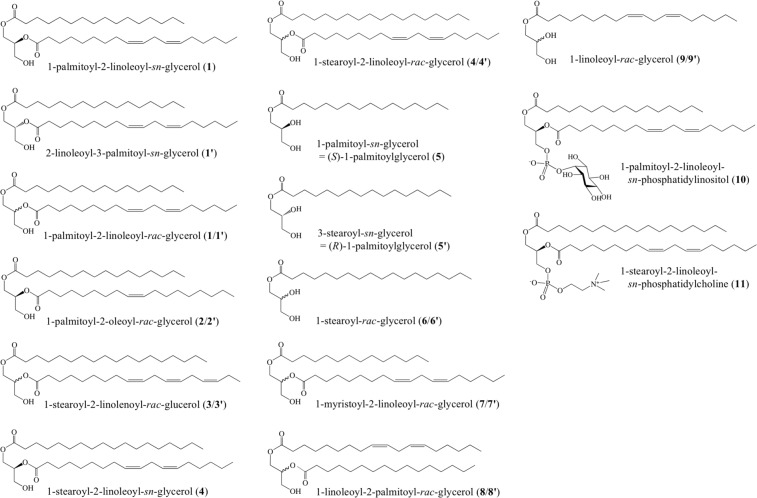
Chemical structures of diacylglycerols (DAGs) and related glycerides that were characterised, synthesised, or examined in this study. Compounds **1**–**4**, **7**, and **8** are DAGs, whereas **5**, **6**, and **9** are MAGs obtainable as synthetic intermediates. Compounds **10** and **11** are phospholipids. Compounds with a simple compound number denotes its natural (*S*)-type lipid, while a compound number with prime as its artificial (*R*)-type lipid. Compounds with number/number with prime are racemic mixture synthesised in this study.

When HIF-1 was treated with lipase under moderate digestion conditions, FD-MS of the reaction products showed two groups of compounds as hydrolyzed fatty acids. The parent ion at *m/z* 280 ([M]^+^, 40%) was consistent with linoleic acid. The second major ion detected at *m/z* 256 ([M]^+^, 35%) was identified as palmitic acid, and the third at *m/z* 284 ([M]^+^, 24%) was identical to stearic acid. The second mass chromatographic peak consisted of two major parent ions at *m/z* 331 ([M + 1]^+^, 53%) and *m/z* 359 ([M + 1]^+^, 100%), which were identical to those of palmitoylglycerol and stearoylglycerol, respectively (Fig. 3c). As the lipase used in this study was a mixture of isozymes from *Candida rugosa*, in which *sn*-2-regioselective lipase A is predominant^31^, the acyl group at the C2 position is released prior to the C1-acyl group in this reaction. In DAG molecules having two different fatty acid chains, the most saturated acyl group is generally found at the *sn*-C1 position^32,33^. The major DAGs in the HIF-1 were thus predicted to be 1-palmitoyl-2-linoleoyl*-sn*-glycerol (**1**), 1-palmitoyl-2-oleoyl*-sn*-glycerol (**2**), 1-stearoyl-2-linolenoyl*-sn*-glycerol (**3**), and 1-stearoyl-2-linoleoyl*-sn*-glycerol (**4**), since two monoacylglycerols from the partly digested product obtained by the treatment with lipase were tentatively identified as 1-palmitoyl-*sn*-glycerol (**5**) and 1-stearoyl-*sn*-glycerol (**6**) (Fig. 4).

Result of the sandwich assay for some DAGs of **1**-homologs and related compounds including *sn*-acyl positional isomer 1-acylated MAGs together with enantiomeric isomers of 1-palmitoyl-2-linoleoylglycerol (**1** and **1′**) and its racemic mixture (**1**/**1′**) are shown here. HIF-like activity is evaluated as follows: (++), the hormogonia induced are dispersed throughout the microscopic field at a high density and collapsing the filamentous cell aggregates into hormogonia (often more than 100 hormogonia per mm^2^); (+), significant numbers of hormogonia are dispersed, but the area where hormogonia dispersed is limited within a part of the microscopic field, together with less collapsion of the filamentous cell aggregates; (±), hormogonia are visible, but are countable due to very low density, or some of the filamentous cell aggregates near the ridge of the paper discs swell as the primary stage of the filamentous aggregate collapsion; and (−), no hormogonia is visible in the microscopic field. (NT), not tested.

### Activity of chemically synthesised DAGs and related compounds

**Figure 5:**
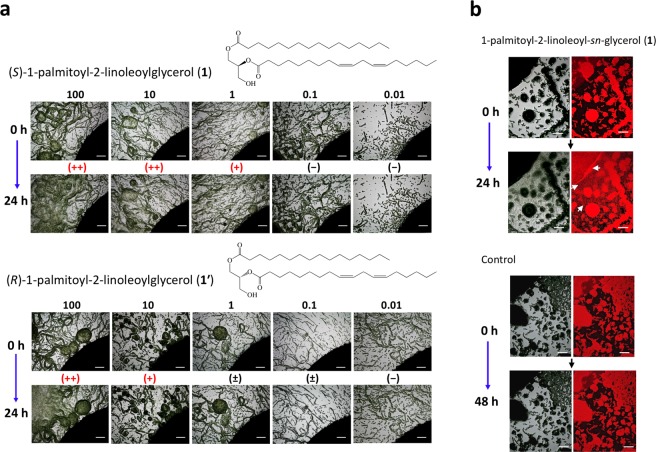
Quantitative and qualitative paper disc assay for hormogonium induction on the filamentous cell aggregates of *Nostoc* sp. strain Yaku-1 by treatment with both enantioisomers (**1** and **1′**) of 1-palmitoyl-2-linoleoylglycerol. (**a**) Using enantiomeric isomers of 1-palmitoyl-2-linoleoylglycerol (**1** and **1′**), the contribution of chirality at the C-2 position to hormogonium-inducing activity of DAGs is examined in the range from 0.01 to 100 nmol/disc. (**b**) For the sandwich assay, cell aggregates are spread on BG-11_0_-G medium in a 12-well polystyrene dish. Arrows in the autofluorescence image of hormogonia induced by **1** (100 nmol/disc) show significant alignment of the hormogonia bundle for direction of the paper disc. Incubation for the treatment with **1** was performed for 24 h at 23 °C, while control was for 48 h. All the scale bars represent 500 µm.

On the basis of our identification of a HIF from the coralloid roots of *C*. *revoluta* as **1**, we chemically synthesised 1-palmitoyl-2-linoleoyl-*sn*-glycerol (=(*S*)-1-palmitoyl-2-linoleoylglycerol, **1**), and the synthesised **1** showed pronounced HIF-like activity at 1 nmol/disc (0.6 μg/disc) (Fig. 5a, Table 1, Supplementary Figs S4–S6). We also chemically synthesised 2-linoleoyl-3-palmitoyl-*sn*-glycerol (=(*R*)-1palmitoyl-2-linoleoylglycerol, **1′**) and 1-palmitoyl-2-linoleoyl-*rac*-glycerol **(1**/**1′**, Supplementary Fig. S7) to examine their relative activity as an HIF-like substance. Compound **1′** was active equivalent to **1** at 1–10 nmol/disc, but at 100 nmol/disc, the activity of **1′** was not observed around the ridge of the paper disc (Fig. 5a). On a BG-11_0_-based 1.2% gellan gum plates, both enantiomers showed obvious activities as the HIF even at 0.1 nmol/disc (Table 1 and Supplementary Fig. S8). In contrast, **1**/**1′** was less active than **1** or **1′** at 100 nmol/disc, but stably active at l and 10 nmol/disc (Supplementary Fig. S9). In addition, compound **1** showed a clear activity as a chemoattractant for the hormogonia induced by **1** itself (white arrows in Fig. 5b).

**Table 1:** Hormogonium-inducing factor (HIF)-like activities of diacylglycerols (DAGs), monoacylglycerols (MAGs), and phospholipids (PGs) structurally related to 1-palmitoyl-2-linoleoyl-*sn*-glycerol.

Compound (glyceride)	Compound/disc (molar amount of test compound)				
	1 × 10^−7^ (100 nmol)	1 × 10^−7^ (100 nmol)	1 × 10^-−9^ (1 nmol)	1 × 10^−10^ (100 pmol)	1 × 10^−11^ (10 pmol)
(*S*)*-*1-palmitoyl-2-linoleoylglycerol (**1**)	++	++	+	−	−
(*R*)*-*1-palmitoyl-2-linoleoylglycerol (**1′**)	++^*a*^	+	±	±	−
1-palmitoyl-2-linoleoyl-*rac*-glycerol (**1/1′**)	+	+	+	±	−
1-palmitoyl-2-oleoyl-*rac*-glycerol (**2/2′**)	++^*b*^	+^*b*^	+^*b*^	±	−
1-stearoyl-2-linolenoyl-*rac*-glycerol (**3/3′**)	+	+	±	−	−
1-stearoyl-2-linoleoy-*sn*-lglycerol (**4**)	+	+	+	−	−
1-myristoyl-2-linoleoyl-*rac*-glycerol (**7/7′**)	+	+	±	±	−
1-linoleoyl-2-palmitoyl-*rac*-glycerol (**8/8′**)	±	±	±	−	NT
(*S*)*-*1-palmitoylglycerol (**5**)	−	−	−	NT	NT
(*R*)*-*1-palmitoylglycerol (**5′**)	−	−	−	NT	NT
1-stearoyl-*rac*-glycerol (**6/6′**)	−	−	−	NT	NT
1-linoleoyl-*rac*-glycerol (**9/9′**)	−	−	−	NT	NT
1-palmitoyl-2-linoleoyl-*sn*-phosphatidylinositol (**10**)	−	−	−	NT	NT
1-stearoyl-2-linoleoyl-*sn*-phosphatidylcholine (**11**)	−	−	−	NT	NT
(*S*)*-*1-palmitoyl-2-linoleoylglycerol (**1**)^*c*^	NT	NT	++	++	NT
(*R*)*-*1-palmitoyl-2-linoleoylglycerol (**1′**)^*c*^	NT	NT	++	++	NT

^*a*^Hormogonium-induction not near side (0–2 mm) but only far side (2 mm or more) from the edge of the paper discs which compound **7/7′** has been charged.

^*b*^Formation of pronounced hormogonium bundle.

^*c*^Bioassay on BG-11_0_-based 1.2% gellan plates, instead of 1.2% agar plates used for other test compounds shown above.

As three more constituents in HIF-1, 1-palmitoyl-2-oleoyl-*sn*-glycerol (**2**), 1-stearoyl-2-linolenoyl-*sn-*glycerol (**3**), and 1-stearoyl-2-linoleoyl-*sn*-glycerol (**4**) were also detected. Both compounds **2** and **3** showing a parent ion at *m/z* 594 and a protonated parent ion at *m/z* 619 respectively were chemically synthesised as racemic DAGs (**2**/**2′** and **3**/**3′**), whereas the fourth major constituent at *m/z* 620 (Fig. 3b), predicted to be **4**, was commercially available. In the hormogonium induction assay (sandwich assay) using enantioselectively synthesised **1** as a positive control, some of the synthesized or commercially available DAGs, commercially available **4** and the racemic mixtures **1**/**1′**, 1-palmitoyl-2-oleoyl-*rac*-glycerol (**2**/**2′**), **3**/**3′**, and 1-myristoyl-2-linoleoyl-*rac*-glycerol (**7**/**7′**) showed HIF activity. Among them, racemic mixture **2/2′** showed the most active hormogonium induction equivalent to enantioselectively synthesised **1**, and the hormogonia induced by **2/2′** uniquely formed thick bundles of the hormogonia (Supplementary Fig. S9). In contrast, 1-linoleoy-2- palmitoyl-*rac*-glycerol (**8**/**8′**), an acyl position isomer of **1/1′**, was almost inactive even at 100 nmol/disc (Table 1).

In addition, monoacylglycerols (MAGs) **5**, **5′**, **6/6′**, and **9/9**′, obtainable as synthetic intermediates of synthesized **1**, **1′**, **3/3′**, and **8/8′** respectively (Supplementary Figs. S4 and S5), were also examined. All of the MAGs thus examined were inactive (Table 1 and Supplementary Fig. S10). In addition, neither simple fatty acids (e.g., palmitic acid, oleic acid, linoleic acid, and α-linolenic acid) nor a triglyceride (e.g., 1,2,3-trilinolenoylglycerol) were active in our bioassay (data not shown). Similarly, neither 1-palmitoyl-2-linoleoyl-*sn*-phosphatidylinositol (**10**) nor 1-stearoyl-2-linoleoyl-*sn*-phosphatidylcholine (**11**) had HIF activity, even at the highest doses (100 nmol/disc) in the sandwich assay.

However, after overnight treatment of **10** with phospholipase C in phosphate buffer (pH 7.0) at room temperature, the small amount of **1** was obtained by preparative TLC (Supplementary Fig. S11a) and confirmed by ^1^H-NMR (Supplementary Fig. S11b). Compound **1** thus obtained from **10** showed a pronounced HIF-like activity at 20 µg (approximately 1.7 µmol)/disc (Supplementary Fig. S12). In addition, crude lipids extracted from the pure-cultured Nostocean cyanobacteria did not show any HIF activity at 10 and 100 µg/discs (data not shown).

**Figure 6:**
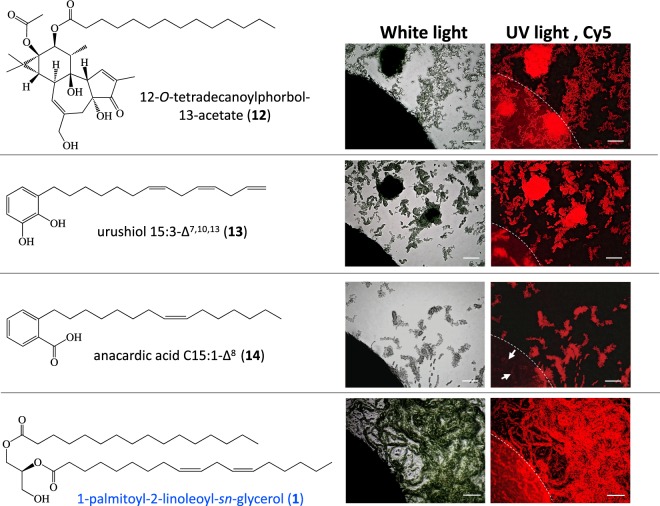
Bioassay for qualitative hormogonium induction of protein kinase C (PKC) activators or skin irritants toward the filamentous cell aggregates of *Nostoc* sp. strain Yaku-1. HIF-like activity of the PKC activators or skin irritants (**12**, **13**, and **14**) are examined at 100 nmol/disc, using 1-palmitoyl-2-linoleoyl-*sn*-glycerol (**1**) as the reference compound. Incubation was performed for 48 h at 23 °C. Arrows in autofluorescence image of **12**-treated Yaku-1 strains using Cy5 filter shows weak hormogonium induction, whereas arrows in autofluorescence image of **14**-treated filamentous Yaku-1 cells have lost red fluorescence under the paper disc, of which edge is indicated with curving broken line. Scale bar represents 500 µm. Disappearance of chlorophyll a under the paper disc is due to lethal toxicity of **14** against cyanobacteria (also see Supplementary Fig. S13).

Activity of the chemical skin irritants 12-*O*-tetradecanoylphorbol-13-acetate (**12**), urushiol C15:3-Δ^7,10,13^ (**13**), and anacardic acid C15:1-Δ^8^ (**14**) was also examined in the sandwich assay (Fig. 6 and Table 2)^34–36^. We found that compounds **12** and **13** showed no HIF-like activity in the range 0.1–10 nmol/disc. Although compound **12** showed any HIF-like activity around the paper disc, this compound at 100 nmol/disc promoted hormogonia formation under the paper disc (white arrows in **12** in Fig. 6). In contrast, compound **14** showed HIF-like activity at 1 nmol/disc, but at higher concentrations (10 and 100 nmol/disc), this compound showed bacteriocidal activity, resulting in discoloration of the cyanobacteria within 48 h (white arrows in **14** in Fig. 6 and Supplementary Fig. S13). Conversely, at 1 nmol/disc, **14** induced differentiation of filamentous cells into hormogonia within 48 h. The lethal toxicity of **14** against filamentous *Nostoc* sp. strain Yaku-1 was nullified by esterification at the carboxy group with methyl and other alkyl alcohols^37^.

**Table 2:** Hormogonium-inducing factor (HIF)-like activities of protein kinase C (PKC) activators or skin irritants.

Compound (glyceride)	Compound/disc (molar amount of test compound)^*a*^				
	1 × 10^−7^ (100 nmol)	1 × 10^−7^ (100 nmol)	1 × 10^−9^ (1 nmol)	1 × 10^−10^ (100 pmol)	1 × 10^−11^ (10 pmol)
12-*O*-tetradecanoylphorbol-13-acetate (**12**)	±^*b*^	−	−	−	−
urushiol C15:3-Δ^7,10,13^ (**13**)	−	−	−	−	−
anacardic acid C15:1-Δ^8^ (**14**)	LT^*c*^	LT^*c*^	+	−	−
(*S*)-1-palmitoyl-2-linoleoylglycerol (**1**)	++	++	+	−	−

^*a*^Bioassay on BG-11_0_-based 1.2% gellan plates, instead of 1.2% agar plates used for other test compounds shown above.

^*b*^Hormogonium-induction not around the paper disc (0–0.5 mm) but slightly under the paper disc, on which compound **12** had been charged.

^*c*^Lethally toxic. Cyanobacterial cells under the paper discs, on which compound **14** had been charged, are missing the red autofluorescence of chlorophyll a, observed only in the viable vegetative cells of the Nostocean cyanobacteria.

Activity of the PKC activators or skin irritants in hormogonium induction from Nostocean filamentous cells is investigated at 100 nmol/disc using the same bioassay method as used for diacylglycerols (DAGs). HIF activity is evaluated as described in the foot note of Table 1. LT means lethally toxic under the paper disc (shown in response to compound **14**).

## Discussion

### Hormogonium-inducing factor

In the plant–microbe interactions characterizing symbiotic associations, the compounds that function as communication signals are often simple monosaccharides, particularly arabinose and glucose easy to biosynthesise^26,38^, or produced from a primary metabolite as common secondary metabolites, such as pigments^39^. As an example of fatty acids or lipids functioning as chemical communication signals, DAGs converted from diacyl-*sn*-phosphatidylinositols by phospholipase C are widely accepted as signaling compounds^40,41^. The DAGs released remain in the membrane and function as protein kinase C activators^42,43^. In plants, several biological activities of DAG endogenously produced from diacyl-*sn*-phosphatidylinositol have been reported^44,45^.

Unlike intracellular signal transduction in DAG-mediated pathways, another important cell-to-cell signaling system involving a lipid component is 1-oleoyl-lysophosphatidylcholine-mediated activation of the phosphate-transporter genes *StPT3* and *StPT4*^41^. Provision of polyphosphate is one of the major contributions of arbuscular mycorrhizal symbionts to their host plants, and 1-oleoyl-lysophosphatidylcholine is a key signaling molecule in this symbiotic process.

In our current study, all the 1-acylated MAGs **5**, **5′**, **6/6′**, and **9/9′**, the racemic mixture **8/8′**, and the diacyl phospholipids (**10** and **11**) were inactive in the sandwich assay. Hence, we predicted key factors of DAGs active as HIF as follows: (**1**) unsaturation of the fatty acid at the C1-position of glycerol moiety prevents HIF-like activity of DAGs, (**2**) lacking acylation at the C2-position also lost the activity, and (**3**) phosphorylation at C3 position also leads to loss of the activity. Probably, HIF-like activity of *sn*-1,2-DAGs, particularly those of **1** and **2**, that are mainly released from the roots of *C*. *revoluta* but rarely contained in *Nostoc* cyanobacteria, is a functionality newly found in the naturally occurring lipid^31,46,47^. Indeed, Peramuna and Summers have reported that lipid body of *N*. *punctiforme* richly contains triacylglycerols and α-tocopherol but is poor in DAGs^48^.

We predicted key factors of DAGs active as HIF as follows: (**1**) unsaturation of the fatty acid at the C1-position of glycerol moiety prevents HIF-like activity of DAGs, (**2**) lacking acylation at the C2-position leads to loss of the activity, (**3**) phosphorylation at C3 position also prevents the activity, and (**4**) both host plants and the symbionts are possible sources of DAGs, but cyanobacteria are poor in the active DAGs^49^.

### Link between DAGs and HIF-like activity

In general, normal-type *C*. *revoluta* roots and *Nostoc* cyanobacteria contain phosphatidylcholine as the major lipid components. Conversely, it has been reported that, under conditions of severe nitrogen deprivation, the transcriptional level of membrane-bound phospholipase C increases in some plants, including *Arabidopsis*, thereby releasing phosphocholine and DAGs into the cytosol and plasma membrane, respectively^50^. As wild *C*. *revoluta* grows in nutrient-poor soils under less competition, deficiencies in both phosphorus and nitrogen may lead to releasing DAGs. Indeed, decomposing coralloid roots of *C*. *revoluta* are often found in soil. When *C*. *revoluta* recovers phosphorus and nitrogen from phosphatidylcholine originated either from the host roots or from the symbionts, releasing excessive DAGs from the biomembranes would be an acceptable event. Thus, the functionality of DAGs toward filamentous *Nostoc* cells newly found assists effective symbiotic establishment of *Nostoc* sp. to *C*. *revoluta* under nitrogen-starvation.

As DAGs are not species-specific HIFs but are widely distributed primary metabolites in plants and cyanobacteria, significance of such signaling compounds is still unknown. Coralloid roots, often indicating smooth branching at the coralloid root tip, also develops new precoralloid root from the base of the coralloid roots to develop the symbiotic organ and allow more effectively the infection of other *Nostoc* strains^51^. Due to this system, coralloid root can grow and develop much faster than the tip branching. Precoralloid root probably accepts new Nostocean cyanobacteria from the bulk soil, because algal zone in the mature coralloid root is separated from newly developing coralloid roots at the base. Hence, discovery of DAGs as potent HIF from the extract of coralloid roots would be reasonable, if the precoralloid roots actively release DAGs as HIF. Indeed, we have searched HIF from *C*. *revoluta* leaf litters, and together with some polyphenols uniquely contained in the leaf litters, fraction containing DAGs also showed active hormogonia-inducing activity. Thus, DAGs are more general activator rather than host-specific HIFs. We will report such host-specific HIF of *C*. *revoluta*, elsewhere.

## Methods

### General

Unless otherwise stated, commercial chemicals of the highest purity were used without further purification. Thin-layer chromatography was performed using Merck silica gel TLC 60 F_254_ (Merck, Darmstadt, Germany) and sprayed with vanillin-sulphuric acid reagent (5% vanillin in 1% conc. sulphuric acid/ethanol). Silica gel column chromatography was performed using silica gel 60 N (spherical, neutral) (Kanto Chemicals, Tokyo, Japan) or Wako gel C200 (Wako, Osaka, Japan). The DAG 1-stearoyl-2-linoleoyl-*sn*-glycerol (**4**), used as a reference compound, and two complex lipids, sodium salts of 1-palmitoyl-2-linoleoyl-*sn*-phosphatidylinositol (**10**) and 1-stearoyl-2-linoleoyl-*sn*-phosphatidylcholine (**11**), were purchased from Funakoshi (Tokyo, Japan). Three further chemicals were purchased as protein kinase C (PKC) activators or skin irritants: 12-*O*-tetradecanoylphorbol-13-acetate (**12**) from Wako, urushiol C15:3-Δ^7,10,13^ (**13**) from Funakoshi, and anacardic acid C15:1-Δ^8^ (**14**) isolated from the fruits of ginkgo (*Ginkgo biloba*), according to the modified method reported by Begum *et al*.^52^.

^1^H NMR spectra were measured in CDCl_3_ solution using a JEOL JNM-EX270 spectrometer. Other chemicals, including commercial media, used in this study are described in the text along with the manufacturer details. Chemical shifts were recorded in *δ* ppm using tetramethylsilane as the internal standard. Coupling constants (*J*) are given in Hertz. Mass spectra were acquired using EI (electron impact) and FD (field diffusion) ionization techniques using a JEOL JMS-SX102A mass spectrometer (JEOL, Tokyo, Japan) in EI- and FD-MS respectively.

### Site description and bacterial source

Sampling sites were Anbo district, Yakushima Island (30°18′44.07′′N, 130°38′18.84′′E), and Kunigami district, Tanegashima Island (30°48′50.26′′N, 131°04′01.18′′E), in Kagoshima Prefecture, Japan. The *Nostoc* sp. isolate used in this study was isolated from the coralloid root of a cycad specimen growing in Anbo district.

### Media

Winogradsky’s medium, which contains no nitrogen sources, was solidified with 1.5% gellan gum (Wako, Osaka, Japan)^29,53^, for plate culture or 0.3% soft gelled gellan for pre-culture of filamentous *Nostoc* sp. and subsequent impregnation into solid medium for hormogonium induction assays. Isolation of the Nostocean cyanobacteria was performed on plates of 1.5% agar containing Winogradsky’s mineral solution (pH 6.2) or BG-11_0_ (nitrogen-free BG-11 medium, pH 7.0) solution^54^, both of which were without supplemented sugar to avoid contamination from heterotrophic bacteria or fungi. The resulting plates were incubated under white LED light (16-h lighting, 2,000–5,000 *lux*) at 25 °C. The medium used is described in the text or the figure legends.

### Isolation of bacteria

The *Nostoc* sp. strain Yaku-1 used in this study was an isolate from the coralloid root of a cycad (*Cycas revoluta*) growing in Yakushima Island, Kagoshima Prefecture, Japan, sampled in 2010^37^. A coralloid root tip (3–5 mm long) stored in 10% glycerol at −80 °C, was surface-sterilized with 2% hypochlorinate solution for 30 s and then rinsed several times with sterile water. The symbiotic cyanobacteria are located between the outer and inner cortex cell layers. From the root tip, tissues containing green-colored intercellular spaces were cut out with a sterile blade as a thin fragment of 2 mm × 2 mm in size. This excised tissue was crushed in a 0.5-mL Eppendorf tube using a flame-sterilized spatula, and a portion of the resulting crushed tissues was streak-cultured on Winogradsky’s mineral solution-based 1.0% agar supplemented with 0.005% yeast extract (MWA), or nitrogen-free BG-11_0_-based 1.5% gellan (BG-11_0_G), each containing 0.2% sucrose. Using a nichrome wire loop, some of the blue-green-colored single colonies were streaked on the plates, which were then incubated at 23 °C for 2 weeks under a 16-h photoperiod. Selected deep green-colored colonies that were clearly apparent within 1 to 2 weeks were repeatedly purified (five times) by streak-culturing on plates.

### Identification of the isolated *Nostoc* sp. strain Yaku-1 based on sequence homology of the 16S rRNA gene

Genomic DNA of *Nostoc* sp. strain Yaku-1 was extracted using Isoplant II (Nippon Gene, Toyama, Japan), and the resulting DNA was subjected to PCR amplification using the *27F*/*1492R* universal primer pair for the 16S rRNA gene^55,56^. The PCR amplicon (1.5 kbp) was sequenced using a direct PCR method with several universal primers for the 16S rRNA gene using an ABI Prism 310 genetic analyzer (Applied Biosystems, Foster City, CA, USA), as described previously^57^. The resulting assembled DNA sequence (1,485 bp, accession no. LC318298) was subjected to a homology search using the DNA Database of Japan (National Center for Biotechnology Information, NCBI). *Nostoc* sp. strain Yaku-1 showed 93% homology with the type-strain of *N*. *punctiforme* ATCC29133^T ^^37^. However, this isolate is involved in the cluster of the type strain *N*. *punctiforme*. Given that Yaku-1 strain was isolated as a symbiont from the coralloid roots of *C*. *revoluta* and formed stable filamentous aggregates on BG-11_0_-based medium, we used strain Yaku-1 for bioassay-guided hormogonium induction.

### Culturing test for *Nostoc* sp. strain Yaku-1 in BG-11_0_ medium

For the pre-culturing of the cyanobacteria as an inoculant, we used BG-11_0_ medium containing 0.2% sucrose (5.8 mM) adjusted to pH 7.2–7.4 with 2 M aq. NaOH, and then filtered through a 0.45-μm OMNIPORE^TM^ PTFE membrane filter (Merck Millipore, Billerica, MA, USA). After adding 1.5% gellan gum powder (Wako, Osaka, Japan), the solution was autoclaved at 121 °C for 15 min, at which point the mixture was shaken well, and cast in plastic petri dishes. *Nostoc* sp. strain Yaku-1 from a stock culture (10% glycerol, −80 °C) was streak-cultured on the plate at 23 °C for 2–4 weeks under 16-h photoperiodic conditions (white LED, 2,000 *lux*). At 2- to 3-week intervals, this purification process was repeated until only filamentous Nostocean cells were observed microscopically and these were subjected to a contamination check on potato-dextrose agar plates. The Nostocean cyanobacteria showed a considerably faster growth response on BG-11_0_ plate than on Winogradsky’s solid medium, and hence we later replaced Winogradsky’s gellan plates with BG-11_0_ medium containing 0.2% sucrose for the following bioassays. Sucrose (0.2%) is expected to show an inhibitory effect on spontaneous differentiation into hormogonia^26^.

### Bioassay protocol

A 20 ml of *Nostoc* sp. strain Yaku-1 pre-cultured in Winogradsky’s mineral solution solidified with 0.3% gellan gum soft gel medium was mixed with 200 ml of Winogradsky’s mineral solution solidified with 0.5% agar previously autoclaved and maintained at 50 °C. Approximately 50–100 of the aggregated filamentous cell colonies per a cm^2^ are the ideal density for impregnated bioassay plates. Paper discs (0.5 mm thick and 8 mm diameter, Advantech Toyo, Tokyo) were autoclaved at 120 °C for 30 min on a wet filter paper, and then oven-dried at 60 °C. After charged crude extract and fractions obtained by following column chromatography on the disc as equivalent amount to those contained in 200 mg fresh coralloid roots as EtOAc or MeOH solutions and air-dried, the assay plate was incubated under the a 16 h-light condition at 30 °C in the plant growth cabinet.

As an improved method, re-incubated colonies of *Nostoc* sp. strain Yaku-1 fast growing on BG-11_0_ gellan plate greater than 5 mm in diameter were collected on an oven-sterilized stainless mesh filter (70 mesh/inch). Mass of the cell aggregates was gently disrupted using a sterile spatula so as to allow the passage of aggregates less than 0.5 mm in size. A suspension of the resulting aggregates was briefly centrifuged at 1000 rpm and re-suspended with BG-11_0_ liquid medium to adjust the concentration to 10 ng chlorophyll/mL medium. A portion of the suspension (1−2 mL) was added to 100 mL BG-11_0_ medium with 1.0% gellan gum instead of Winogradsky’s mineral solution solidified with 0.5% agar for the impregnation assay.

For sandwich assay, BG-11_0_ medium was solidified with 0.6% agar or 1.0% gellan gum (w/v), and each medium (2 mL) already autoclaved was poured in each 12-well to allow solidification. A 50 µL portion of the *Nostoc* cell aggregate suspension was spread on the BG-11_0_ solid media in 12-well plate and air-dried. Onto this 12-well plate pre-cultured for 2 weeks, a ø 8 mm paper disc (0.7 mm thick) applied hormogonia-inducing chemical substance was placed, and responses of filamentous cell aggregates as morpho-differentiation into hormogonia just under or around the paper disc were observed, using a fluorescent microscope BioRevo (Keyens, Osaka, Japan).

### Bioassay-guided purification of hormogonia-inducing factor

In extraction from *C*. *revoluta* coralloid roots (1.08 kg fresh weight), the concentrated MeOH extract formed a tar-like solid. Therefore, this extract was initially partitioned between water and EtOAc (Fig. 1). Both EtOAc- and water-soluble materials were subjected to the paper disc assay for hormogonium induction using 1.0% gellan plates impregnated with small colonies of filamentous *Nostoc* cell aggregates (Fig. 2). The HIF-like active principle found in the EtOAc-soluble fractionated by silica gel column chromatography (F1Fr. 5, 453 mg, by the 3^rd^ bioassay) was further chased by HIF-like activity-based (by the sandwiched bioassay shown in Fig. 2) 2^nd^ silica gel column chromatography. The 2^nd^ silica gel column chromatography of F1Fr-5 with stepwise elution with acetone/toluene yielded an active fraction (F2Fr-10, 134 mg, by the 4^th^ bioassay). Further silica gel column chromatography for all the active fractions F2Fr-10 by isocratic elution with *n*-hexane-CHCl_3_-EtOAc (10:9:1) yield a colorless syrup (active fractions F3Fr-13-20, 100 mg by the 5^th^ bioassay), and the active fractions were re-separated by column chromatography using the same solvent system (*n*-hexane-CHCl_3_-EtOAc, 10:9:1) to yield an active fraction F4Fr-2 (62 mg, by the 6^th^ bioassay). As this fraction contained chlorophyll as a contaminant, 60 mg of F4Fr-2 was finally subjected to silica gel column chromatography with *n*-hexane-EtOAc (4:1), and the active principle (HIF-1, 24 mg, by the 7^th^ bioassay) was obtained as a single spot on TLC (Fig. 3a), and subjected to FD-MS (Fig. 3b) and ^1^H-NMR analyses (Supplementary Fig. S3).

Thus, HIF-1 contained no quenching spot on silica gel TLC 60F-5725, whereas it clearly responded to vanillin-sulfuric acid reagent as a pinkish colored spot. In the hormogonium induction assay, 0.2 µg of the purified mixture per disc showed pronounced induction of hormogonia in the Nostocean filamentous cell aggregates.

### Lipase hydrolysis of HIF-1

Lipase AYS from *Candida rugosa* (Amano Enzyme Inc., Nagoya, Japan) was used for hydrolysis of HIF-1. HIF-1, obtained as a mixture of DAGs (200 mg), was dissolved in a small volume of MeOH (<100 m), then dropped into 10 mL of 50 mM sodium phosphate buffer (pH 7.0) containing 1 mg of the lipase (2 U), and subsequently stirred overnight at room temperature. The reaction mixture was then acidified with 1 M HCl to pH 4.0, and twice extracted with an equal volume of EtOAc. The organic layer dried over anhydrous Na_2_SO_4_ was concentrated and re-dissolved in a small volume of MeOH (50 mL). The product giving a new spot at a smaller *Rf* value than the HIF on silica gel TLC was obtained by preparative TLC (hexane-EtOAc 4:1, ca. 100 mg), and subjected to FD-MS (Fig. 3c).

### Investigation of the HIF-like activity of some PKC activators

Since DAGs that act as HIFs are known to be PKC activators, other representative PKC activators^58^, particularly those originating from plants, were investigated. As low molecular weight PKC activators, we selected 12-*O*-tetradecanoylphorbol-13-acetate (**12**)^34,35^ and urushiol C15:3-Δ^10,13,16^ (**13**)^34,35^. In addition, anacardic acid C15:1-Δ^8^ (**14**), which exhibits a 7-like biological activity toward mammalian cells but shows a different mode of action, was examined due to its activity as a PI3K-dependent stimulator for neutrophils via activation of surface-expressed G protein-coupled sphingosine-1-phosphate receptors^36,59^. These three compounds were subjected to the paper disc assay for hormogonium induction using a range of concentration from 0.1 to 100 nmol/disc.

## Supplementary information

Fig. S1, Fig. S2, Fig. S3, Fig, S4, Fig. S5, Fig. S6, Fig. S7, Fig. S8, Fig, S9, Fig. S10, Fig. S11, Fig. S12, Fig. S13
