# Supplementary material for: Isolation and characterization of 1-palmitoyl-2-linoleoyl-sn-glycerol as a hormogonium-inducing factor (HIF) from the coralloid roots of Cycas revoluta (Cycadaceae)
Source: Sci Rep. 2019 Mar 18;9:4751. doi: 10.1038/s41598-019-39647-8 (PMC6426835; doi:10.1038/s41598-019-39647-8)
Supplement: Supplementary file 1 — Fig. S1, Fig. S2, Fig. S3, Fig, S4, Fig. S5, Fig. S6, Fig. S7, Fig. S8, Fig, S9, Fig. S10, Fig. S11, Fig. S12, Fig. S13 [file 41598_2019_39647_MOESM1_ESM.pdf]

Isolation and characterization of 1-palmitoyl-2-linoleoyl-*sn*-glycerol as a hormogonium-inducing factor (HIF) from the coralloid roots of *Cycas revoluta* (Cycadaceae)

Yasuyuki Hashidoko[1*], Hiroaki Nishizuka[1], Manato Tanaka[1], Kanako Murata[1], Yuta Murai[2], and Makoto Hashimoto[1]

[1] Division of Applied Bioscience, Research Faculty of Agriculture, Hokkaido University, Kita 9 Nishi 9, Kita-ku, Sapporo 060-8589, Japan

[2] Present address: Division of Life Science, Graduate School of Life Science, Hokkaido University, Kita 10 Nishi 8, Kita-ku, Sapporo 060-0810, Japan

Fig. S1. Symbiotic process of *Nostoc* cyanobacteria to recognize host plant, infect with symbiotic part, and differentiate as nitrogen fixer

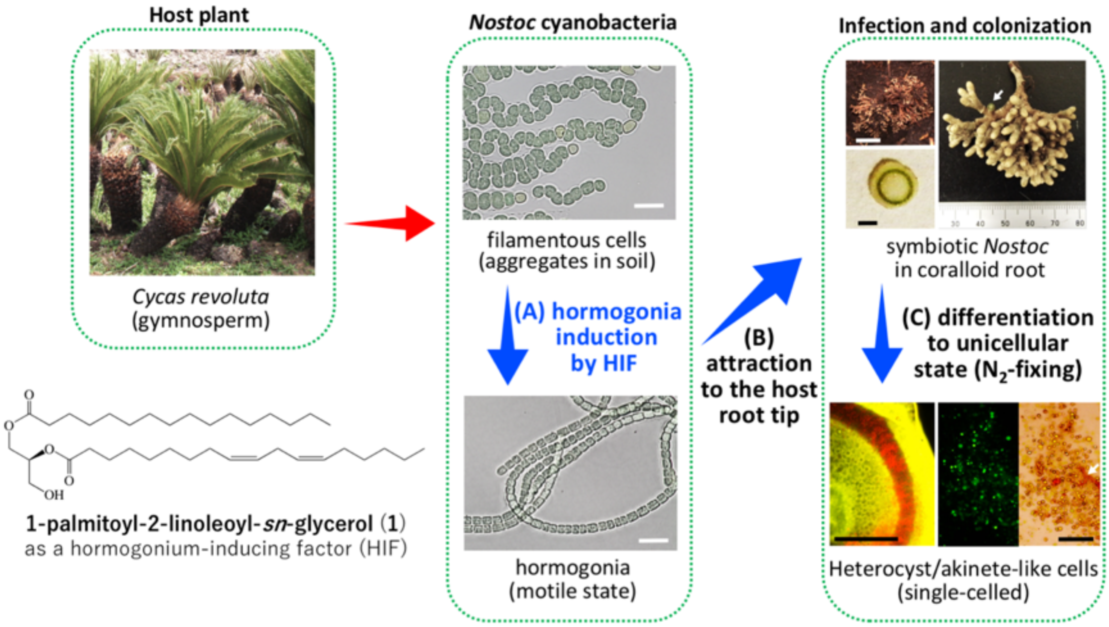

Host plant: *Cycas revoluta* often establishes symbiosis with *Nostoc* cyanobacteria at the coralloid roots. ***Nostoc* cyanobacteria**: To attract the symbiont, *C. revoluta* would produce hormogonium-inducing factor (HIF) and induce immotile filamentous cells of *Nostoc* to a motile form known as hormogonia (**A**). The white bars represent 10 µm. *Nostoc* hormogonia can swim in water in soil or on plant surfaces; thus, their movements can be resulted in attraction to the host plant (**B**). **Infection and colonization**: (Top panels) Such chemoattractant would navigate the motile hormogonium to reach to the tip of pre-coralloid root, where symbiosis with the host plant is developed as mature coralloid root. The white bar represent 5 cm, and the black solid bar represent 1 mm. (Bottom panels) Differentiation of *Nostoc* cyanobacteria to N_2-fixing state would be induced by hypothetical nitrogen-fixing state-inducing factor (**C**). Mature coralloid roots harbor algal endophytes in intercellular spaces known as algal zone, in which the cells elongate radially between inner cortex and outer cortex. (Bottom-left panel) Macrophotograph of the perpendicularly sectioned coralloid root under white light is merged with that under UV light through Cy5 for red autofluorescence of chlorophyll a. Bar represents 0.5 mm. (Bottom-center and right panels) Most of the symbiotic *Nostoc* cyanobacteria are heterocyst-like unicellular, spherical forms in the algal zone, while some filamentous cells also remain (white arrow). The center panel shows bacterial LIVE/DEAD assay using CYTO 9/propidium iodide. The right panel under white light merged with that under UV light through Cy5 is the same view with the photograph at the center panel. Black bar represents 50 µm.

Fig. S2. Hormogonium-inducing activity of an active fraction of an ethyl acetate-soluble in "Sandwiched bioassay"

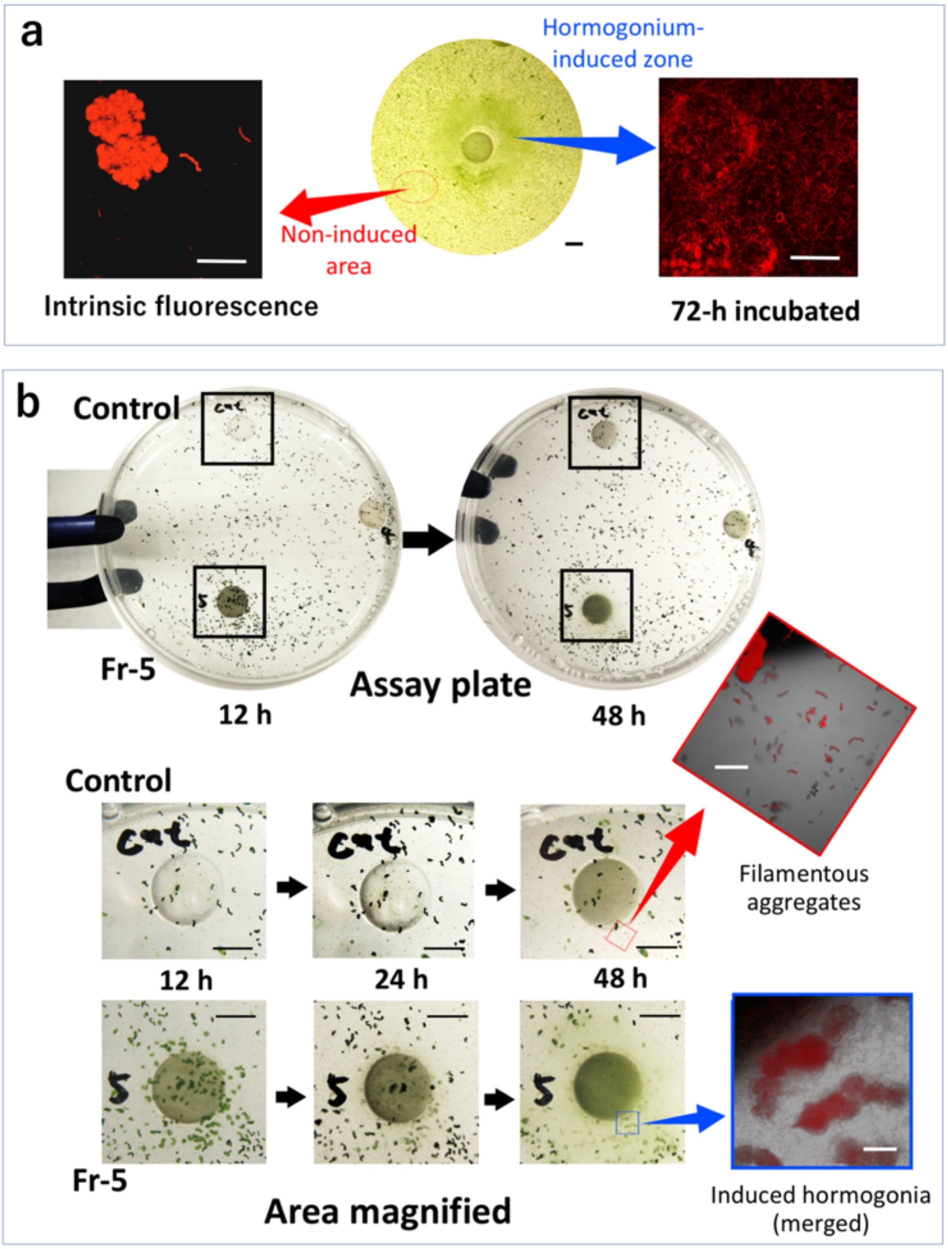

a Impregnated bioassay. Black solid bar is 5 mm, while white solid bars in microscopic photographs are 500 µm. **b** Sandwiched bioassay on a petri dish. This bioassay is examined by the EtOAc-soluble fractionated as active F1Fr-5, as that equivalent to 2 g of coralloid roots. Approximate areas shown by squares in the sandwich assay are magnified as its Cy5-UV or white light. Incubation time is 48 h. Black solid bars in the macro focused photographs represent 5 mm, while white solid bars in microscopic photographs represent 500 µm.

Fig. S3. ^{1}H-NMR spectrum of HIF-1

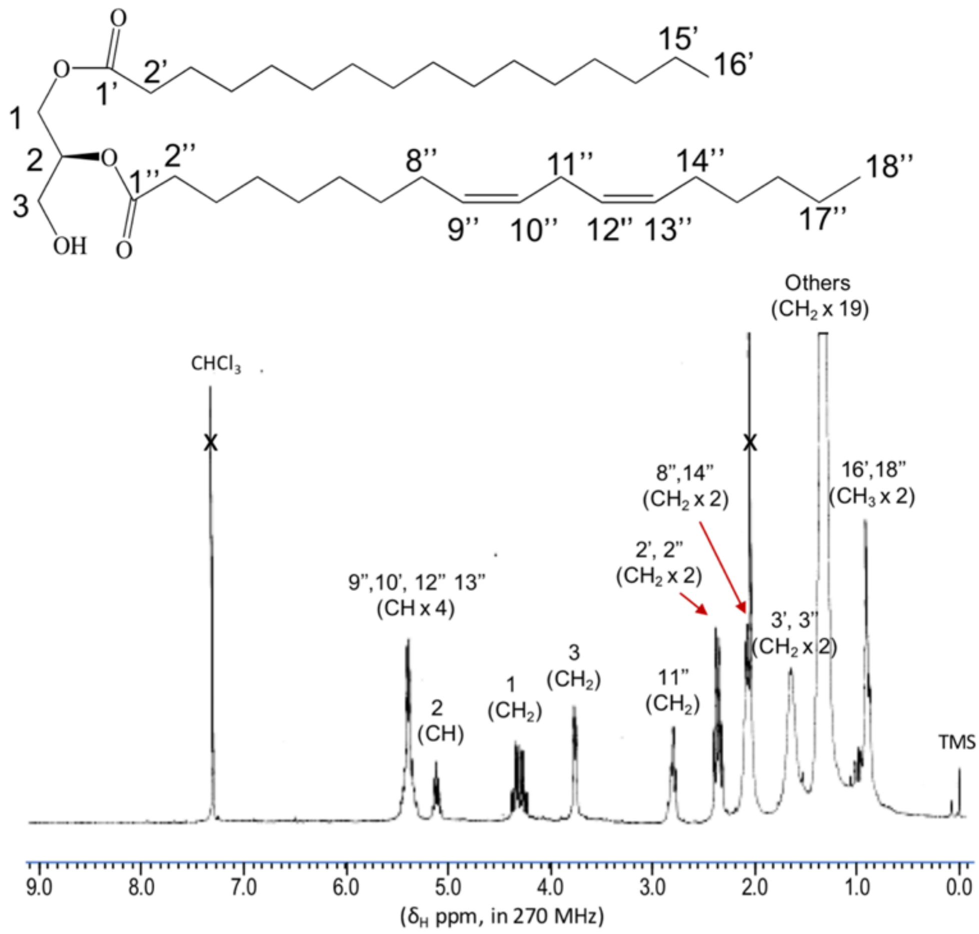

Spectrum was measured in $CDCl_3$ in 270 MHz. HIF-1 was assigned as a mixture of DAGs. From the proton signals, the main compound in HIF-1 was assignable as 1-palmitoyl-2-linoleoylglycerol. To identify stereochemistry for the main component of HIF-1, both 1-palmitoyl-2-linoleoyl-*sn*-glycerol (**1**) and 2-linoleoyl-3-palmitoyl-*sn*-glycerol (**2**) were enantioselectively synthesised. Spectroscopic analysis of HIF-1. ^{1}H NMR (in $CDCl_3$, 270 MHz): 5.35 (ca. 4-5H, br. m, *Z*-olefinic H in linoleoyl group), 5.08 (1H, ddd, *J*=5.6, 5.4, and 4.6 Hz, acylated *O*-CH at C-2 position in glycerol moiety), 4.32 (1H, dd, *J*=11.9 and 5.4 Hz, one of the proton signals acylated *O*-CH_2 at C-1 position in glycerol moiety), 4.23 (1H, dd, *J*=11.9 and 5.6 Hz, another acylated 1-CH_2 in glycerol moiety), 3.73 (2H, br. d, *J*=4.6 Hz, C-3-CH_2 substituted with a free primary OH in glycerol moiety), 2.78 (2H, m, bisallyl CH_2 at C-11″ in linoleoyl group), 2.34 (4H, 2′-CH_2 and 2″-CH_2 in α-position of carbonyl groups), 2.05 (4H, m, 8″-CH_2 and 14″-CH_2 at allyl position in the linoleoyl group), 1.62 (4H, br., 3′-CH_2 and 3″-CH_2 in β-position of carbonyl groups), 1.31 and 1.26 (remaining signals from CH_2 in acyl chains), and 0.89 (6H, terminal CH_3 × 2).

Fig. S4. Process for the enantioselective synthesis of 1-palmitoyl-2- linoleoyl-*sn*-glycerol

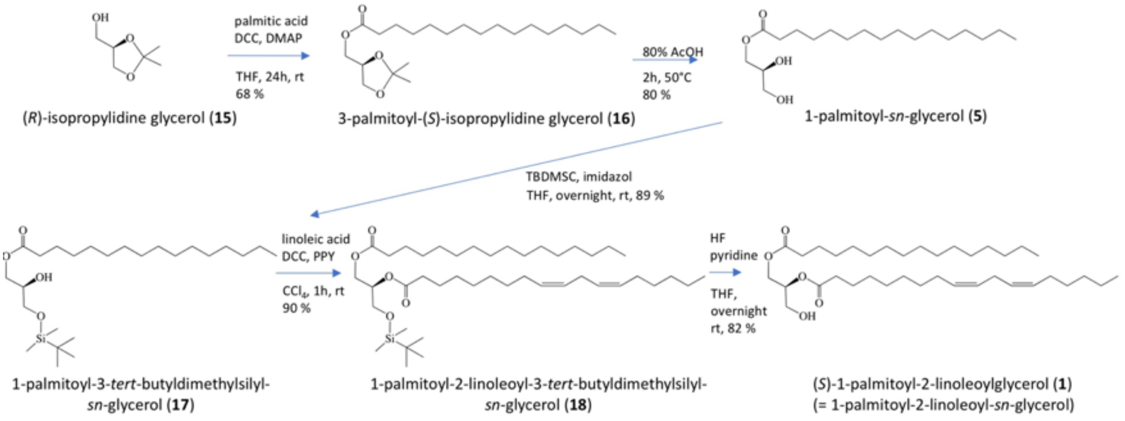

To palmitic acid (0.68 g, 2.65 mmol) dissolved in dry THF (20 mL), DCC (0.54 g, 2.65 mmol), DMAP (0.32 g, 2.65 mmol), and (*R*)-isopropylidine glycerol (**15**, 0.42 g, 3.19 mmol) were added[1]. After stirring for 24 h at room temperature, the reaction mixture was filtered and concentrated. The resulting oil was purified by silica gel column chromatography (hexane:EtOAc, 9:1), and main product **16** was isolated as colorless solid (0.67 g, 68% yield). *3-Palmitoyl-(*R*)-isopropylidine glycerol (**16**)*. FD-MS (*m/z*, %): 371.28 ($[M+H]^+$, 8), 356.25 ($[M-CH_3+H]^+$, 22), and 355.21 ($[M-CH_3]^+$, 100).

Aqueous acetic acid ($AcOH:H_2O$, 80:20, 12 mL) was added to compound **16** (0.55 g, 1.5 mmol), and the mixture was stirred and heated to 50 °C for 2 h. After complete disappearance of **16**, the mixture was cooled down and diluted with EtOAc, followed by washing with water, $NaHCO_3$ solution, and finally with brine. The resulting organic layer was dried over anhydrous Na_2SO_4 and concentrated to yield colorless solid. This solid was recrystallized from EtOAc/hexane to administer 0.39 g (80% yield) of **5** as colorless plates. *1-Palmitoyl-*sn*-glycerol (**5**)*. FD-MS (*m/z*, %): 331.25 ($[M+H]^+$, 100), 299.23 (26), and 238.21 ($[M-C_3H_8O_3]^+$, 14).

TBDMSCl (0.15 g, 1.0 mmol) and imidazole (93 mg, 1.4 mmol) were added to a solution of **5** (300 mg, 0.91 mmol) in THF. After stirring overnight, the reaction mixture yielding colorless precipitates was filtered and dried *in vacuo*. The main product **17** was purified by silica gel column chromatography (*n*-hexane:EtOAc, 6:1) and isolated as colorless oil (360 mg, 89% yield). (*R*)-*1-Palmitoyl-3-*tert-*butyldimethylsilylglycerol (**17**)*. FD-MS (*m/z*, %): 445.35 ($[M+H]^+$, 53) and 387.27 ($[M-C_3H_8]^+$, 100).

A solution of DCC (78 mg, 0.38 mmol) dissolved in CCl_4 (15 mL) was added dropwise to a solution of compound **17** (140 mg, 0.31 mmol), linoleic acid (0.11 mL, 0.35 mmol), and 4-pyrrolidinopyridine (56 mg, 0.38 mmol) in CCl_4 (15 mL). After 1 h stirring at room temperature,

precipitates in the reaction mixture was filtered. The crude product was purified by silica gel column chromatography (*n*-hexane:EtOAc, 30:1) to afford main product **18** as colorless oil (197 mg, 90% yield). *(*R*)-1-Palmitoyl-2-linoleoyl-3-*tert*-butyldimethylsilylglycerol (**18**)*. FD-MS (*m/z*, %): 706.62 ($[M]^+$, 24), 649.54 ($[M\text{-}C_3H_8]^+$, 28), 501.40 (12), 310 (65), and 309 (100).

Hydrogen fluoride pyridine (60 μL, 0.65 mmol) was added to a solution of compound **18** (150 mg, 0.21 mmol) and pyridine (55 μL, 0.65 mmol) in THF (10 mL)[2]. After stirring overnight at room temperature, the reaction mixture was diluted with hexane and washed with H_2O, $NaHCO_3$ solution, and then brine. The resulting organic layer was dried over anhydrous Na_2SO_4 and concentrated. Main product **1** was obtained as pale yellow oil (103 mg, 82%). *(R)-1-Palmitoyl-2-linoleoylglycerol (=1-palmitoyl-2-linoleoyl-*sn*-glycerol, **1**)*. FD-MS (*m/z*, %): 530.50 ($[M+H]^+$, 58), and 592.50 ($[M]^+$, 100). Optical rotation: $[\alpha]^{25}_D$ +0.8 ° (*c*=1, 10 mg of **1** dissolved in 1.0 mL of $CHCl_3$). ^{1}H-NMR spectrum, agreeable with that of HIF-1, is shown in Supplementary Fig. S4.

For enantioselective synthesis of (*R*)-1-palmitoyl-2-linoleoylglycerol (= 2-linoleoyl-3-palmitoyl-*sn*-glycerol, **1'**), synthetic procedure was almost the same, except for starting material (*S*)-isopropylidine glycerol (**15'**). Main product 3-palmitoyl-(*S*)-isopropylidine glycerol (**16'**) was obtained as colorless plates (0.62 g, 63% yield). Compound **16'** (500 mg, 1.35 mmol) was deprotected with aqueous acetic acid to yield 3-palmitoyl-*sn*-glycerol (**5'**, 0.39 g of colorless oil, 88% yield). This monoacylglycerol (300 mg, 0.91 mmol), was *tert*-butyldimethylsilylated at 1-OH to yield (*R*)-1-*tert*-butyldimethylsilyl-3-palmitoyl-*sn*-glycerol (**17'**, 323 mg, 80% yield). At the 2-OH position of **17'** (300 mg, 0.67 mmol), linoleic acid (0.23 mL, 0.74 mmol) was condensed to yield (*R*)-1-*tert*-butyldimethylsilyl- 2-linoleoyl-3-palmitoyl-*sn*-glycerol (**18'**, 420 mg, 88% yield). From compound **18'** (300 mg, 0.42 mmol), *tert*-butyldimethylsilyl group was removed to yield **1'** as pale yellow oil (230 mg, 90%). Optical rotation was $[\alpha]^{25}_D$ −0.8 ° (*c*=1, 10 mg of **1'** dissolved in 1.0 mL of $CHCl_3$).

1. Gunstone, F. D., Harwood, J. L., & Padley, F. B. The Lipid Handbook, Second Edition, Chapman & Hall, Electronic Publishing Division, pp. 366−374 (1994).
2. Nelson, T. D., & Crouch, R. D. Selective deprotection of silyl ethers. *Synthesis* **9**, 1031−1069 (1996).

Fig. S5. FD-MS spectra of enantioselectively synthesised 1-palmitoyl-2-linoleoyl-*sn*-glycerol and its synthetic intermediates

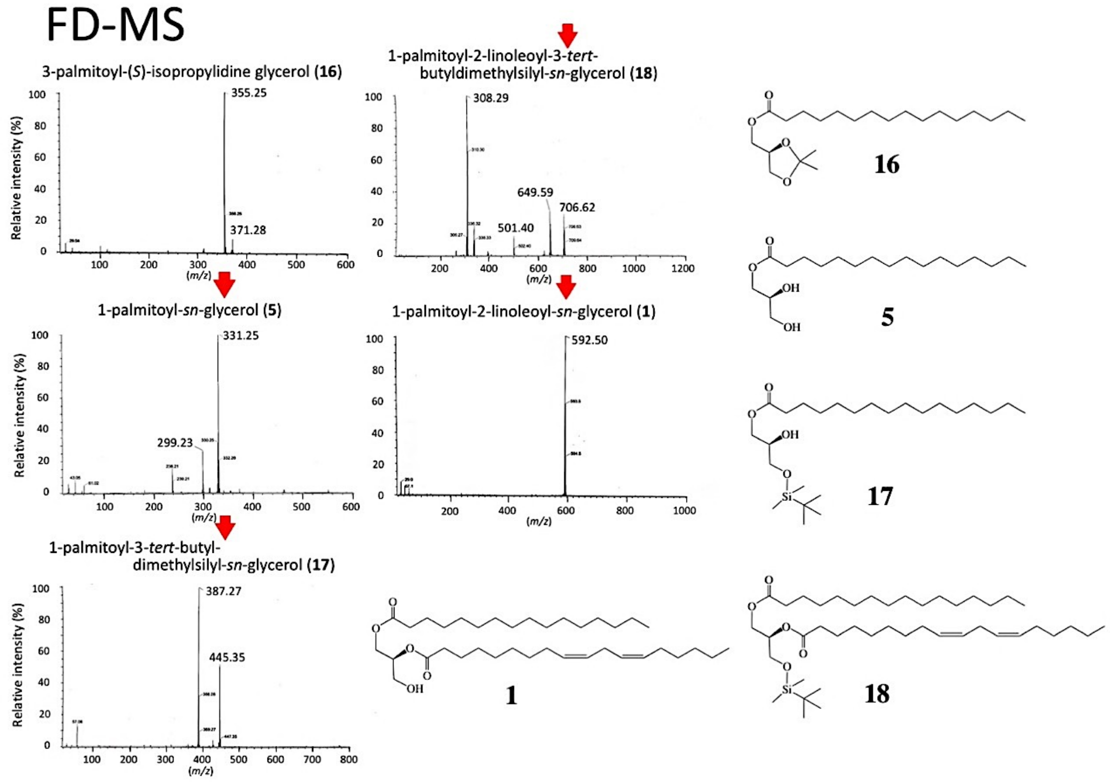

In FD-MS analyses, 3-palmitoyl-(*R*)-isopropylidine glycerol (**16**), (*R*)-1-palmitoyl-*sn*-glycerol (**5**), (*R*)-1-palmitoyl-3-*tert*-butyldimethylsilylglycerol (**17**), (*R*)-1-palmitoyl-2-linoleoyl-3- *tert*-butyldimethylsilylglycerol (**18**), and the final product (*S*)-1-palmitoyl-2-linoleoylglycerol (=1-palmitoyl-2-linoleoyl-*sn*-glycerol, **1**) administered parent ions *m/z* 371.28 ($[M+H]^+$, 8% intensity), 331.25 ($[M+H]^+$, 100%), 445.35 ($[M+H]^+$, 53%), 706.62 ($[M]^+$, 24%), and 592.50 ($[M]^+$, 100%), respectively.

Fig. S6. ^{1}H-NMR spectrum of enantioselectively synthesized 1-palmitoyl-2-linoleoyl-*sn*-glycerol

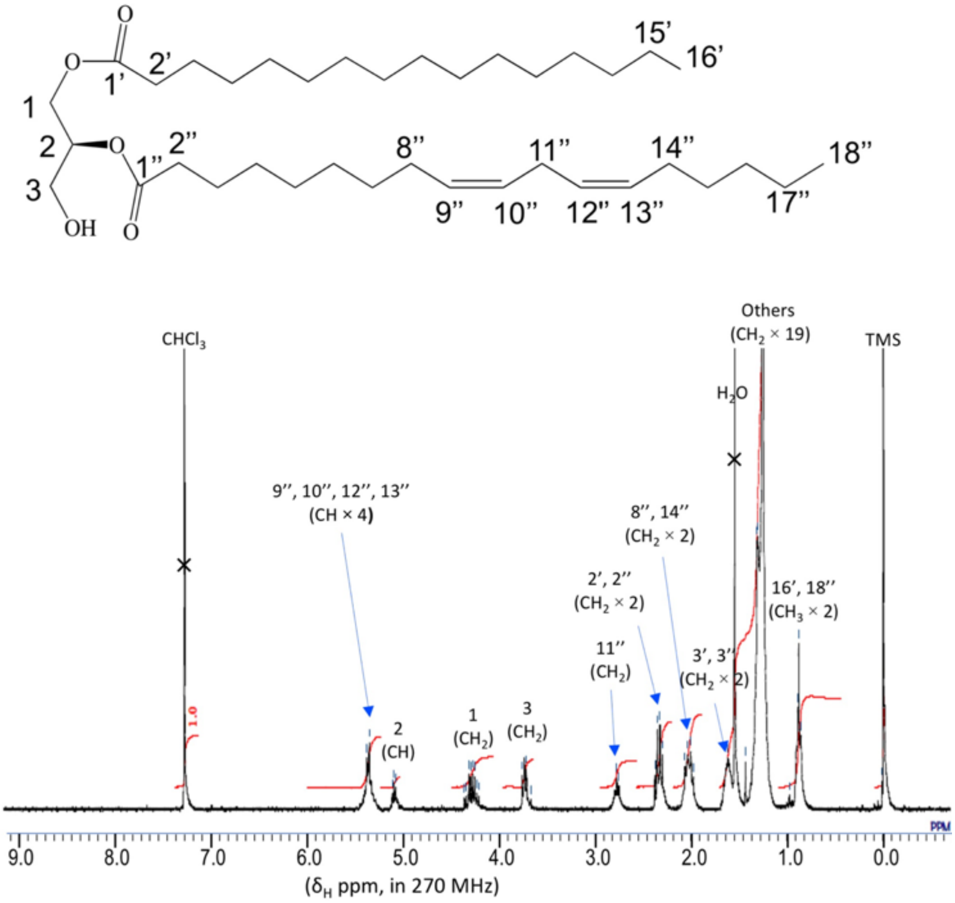

Spectrum of the enantioselectively synthesised 1-palmitoyl-2-linoleoyl-*sn*-glycerol [= (*S*)-1-palmitoyl-2-linoleoylglycerol (**1**)] was measured in $CDCl_3$ in 270 MHz. 5.35 (ca. 4H, br. m, *Z*-olefinic H in linoleoyl group), 5.08 (1H, ddd, *J*=5.6, 5.4, and 4.6 Hz, acylated *O*-CH at C-2 position in glycerol moiety), 4.32 (1H, dd, *J*=11.9 and 5.4 Hz, one of the proton signals acylated *O*-CH_2 at C-1 position in glycerol moiety), 4.23 (1H, dd, *J*=11.9 and 5.6 Hz, another acylated 1-CH_2 in glycerol moiety), 3.73 (2H, br. d, *J*=4.6 Hz, C-3-CH_2 substituted with a free primary OH in glycerol moiety), 2.78 (2H, m, bisallyl CH_2 at C-11″ in linoleoyl group), 2.34 (4H, 2′-CH_2 and 2″-CH_2 in α-position of carbonyl groups), 2.05 (4H, m, 8″-CH_2 and 14″-CH_2 at allyl position in the linoleoyl group), 1.62 (4H, br., 3′-CH_2 and 3″-CH_2 in β-position of carbonyl groups), 1.31 and 1.26 (remaining signals from CH_2 in acyl chains), and 0.89 (6H, terminal CH_3 × 2).

Fig. S7. Process for the racemic synthesis of 1-palmitoyl-2-linoleoyl-*rac*-glycerol

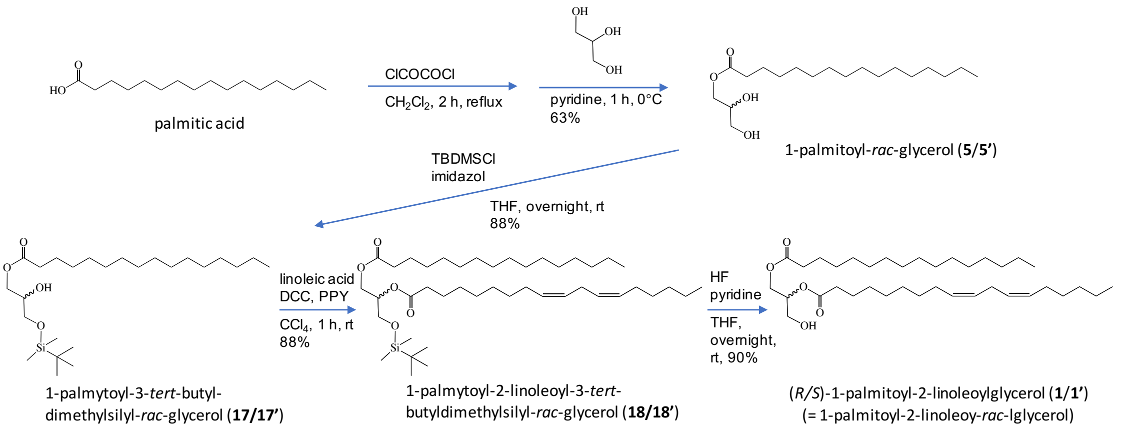

A solution of palmitic acid (10 mmol) and oxalyl chloride (15 mmol) in CH_2Cl_2 (10 mL) was refluxed for 2 h and then evaporated to remove the solvent. The oily residue (palmitoyl chloride) was re-dissolved in CH_2Cl_2 (5 mL), and the solution was then added dropwise to glycerol (50 mmol) dissolved in pyridine (20 mL) stirred at 0°C. The reaction mixture was kept stirring for 1 h at 0 °C, at which point the solution was poured into ice water. Then, the mixture was extracted twice with $CHCl_3$, and the organic layers combined were washed with ice-cold 2M HCl followed by water. After dried over anhydrous Na_2SO_4, the residues obtained were concentrated *in vacuo* and subjected to silica gel column chromatography ($CHCl_3$:MeOH, 19:1). Colorless plates (2.1 g, 63% yield) were obtained as the resulting isolate, 1-palmitoyl-*rac*-glycerol (**5**/**5'**).

Tert-butyldimethylsilyl chloride (TBDMSCl) (0.54 g, 3.6 mmol) and imidazole (300 mg, 4.5 mmol) were added to a solution of compound **17**/**17'** (1.0 g, 3.0 mmol) in THF[1]. After stirring overnight at room temperature, the reaction mixture was filtered to obtain colorless precipitates. The product was purified by silica gel column chromatography (*n*-hexane:EtOAc, 4:1), and main product 1-palmitoyl-3-*tert*-butyldimethylsilyl-*rac*-glycerol (**17**/**17'**) was obtained as a colorless liquid (1.2 g, 88% yield). A solution of DCC (270 mg, 1.3 mmol) in CCl_4 (15 mL) was added dropwise to a solution of compound **14**/**14'** (500 mg, 1.1 mmol), linoleic acid (0.38 mL, 1.2 mmol), and 4-pyrrolidinopyridine (180 mg, 1.2 mmol) in the reaction mixture. After stirring 1 h at room temperature, the reaction mixture yielded a colorless precipitate. The precipitate was filtered, concentrated, and subjected to silica gel column chromatography (hexane:EtOAc, 30:1). The purification afforded main product 1-palmitoyl-2-linoleoyl-3-*tert*-butyldimethylsilyl- *rac*-glycerol (**18**/**18'**) as a colorless oil (699 mg, 88% yield).

Hydrogen fluoride pyridine (200 μL) was added to a solution of compound **18**/**18'** (500 mg, 0.7 mmol) in THF (10 mL), followed by addition of pyridine (170 μL, 2.1 mmol)[2]. After stirring overnight at room temperature, the reaction mixture was diluted with *n*-hexane and then washed with H_2O followed by $NaHCO_3$ solution and brine. The organic layer was dried over Na_2SO_4 and concentrated. The product 1-palmitoyl-2-linoleoyl-*rac*-glycerol (**1**/**1'**) was obtained as a pale

yellowish oil (380 mg, 90%). *1-Palmitoyl-2-linoleoyl*-rac-*glycerol* ***(1/1')***: FD-MS (*m/z*, %): 592.5 ($[M]^+$, 100) and 593.5 ($[M+1]^+$, 58).

1. Corey, E. J. & Venkateswarlu, A. Protection of hydroxyl groups as *tert*-butyldimethylsilyl derivatives. *J. Am. Chem. Soc.* **94**, 6190−6191 (1972).
2. Nelson, T. D., & Crouch, R. D. Selective deprotection of silyl ethers. *Synthesis* **9**, 1031−1069 (1996).

Fig. S8. Hormogonium inducing activity of enantioisomers (1 and 1′) of 1-palmitoyl-2-linoleoylglycerol on 1.2% gellan gum

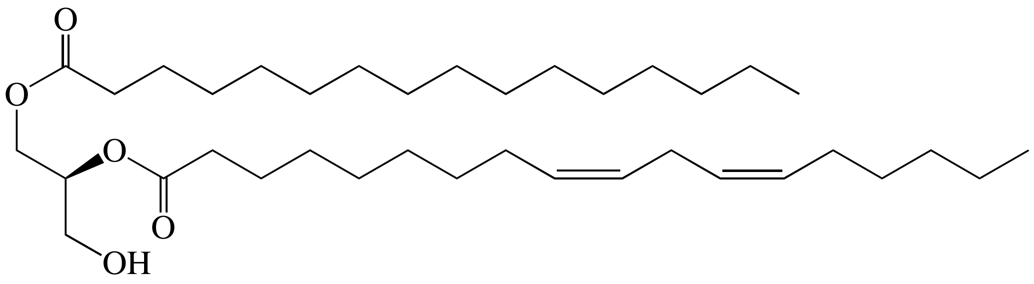

(*S*)-1-palmitoyl-2-linoleoylglycerol (**1**)

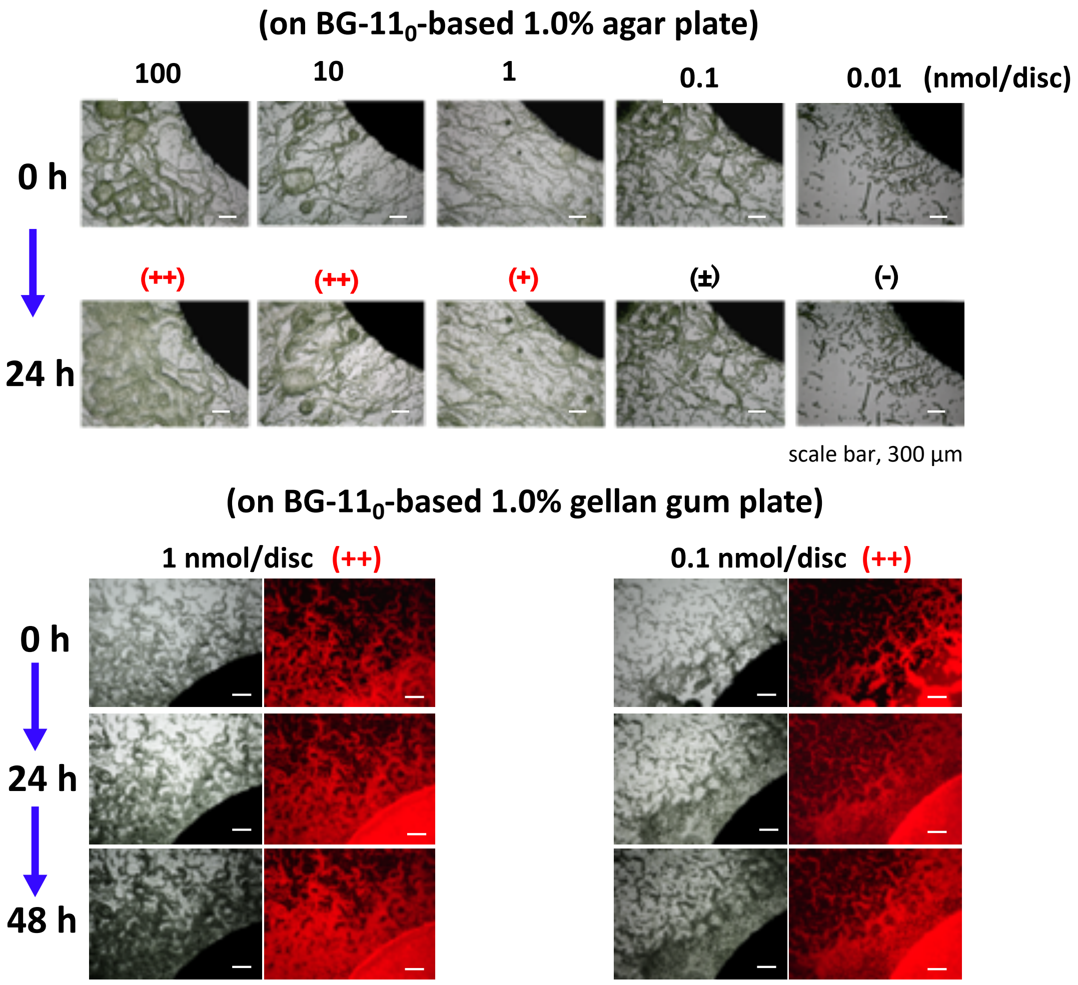

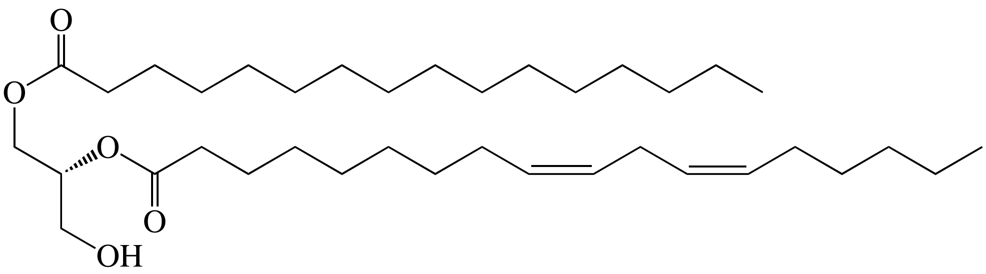

(*R*)-1-palmitoyl-2-linoleoylglycerol (**1′**)

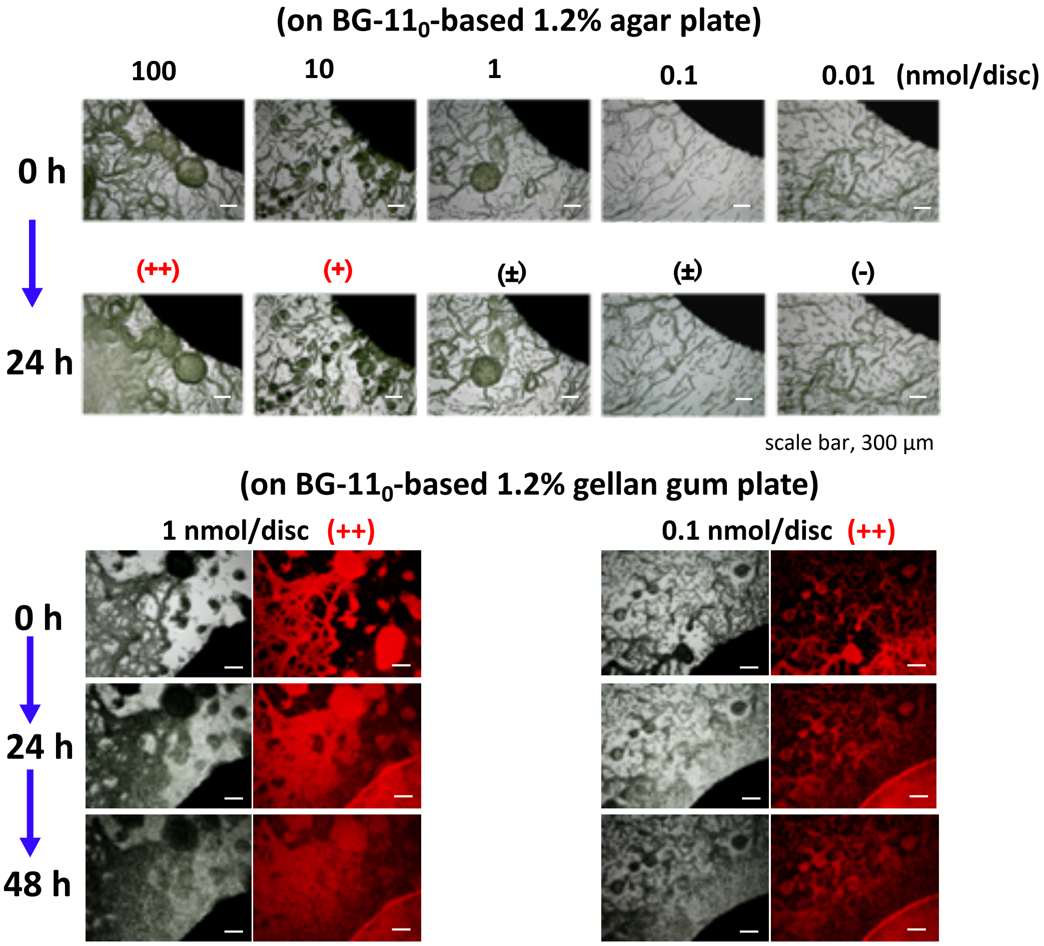

HIF-like activities of (*S*)- and (*R*)-1-palmitoyl-2-linoeoylglycerols (**1** and **1′**) were compared using BG-11$_0$-1.2% agar or gellan gum plate. Hormogonium-inducing activities of **1** (a set of the first panels) and **1′** (the second set of panels) were examined in the range from 0.01 to 100 nmol/disc (panels for BG-11$_0$-based 1.2% agar plate, top panels) and at 0.1 and 1 nmol/disc (on 1.2% gellan gum plates, bottom panels). The latter photographs are under white light (left) and UV light through Cy5 filter (right). Scale bar represents 500 μm. Both of the controls did not show any hormogonia induction.

Fig. S9. Hormogonium-inducing activity of varying DAGs

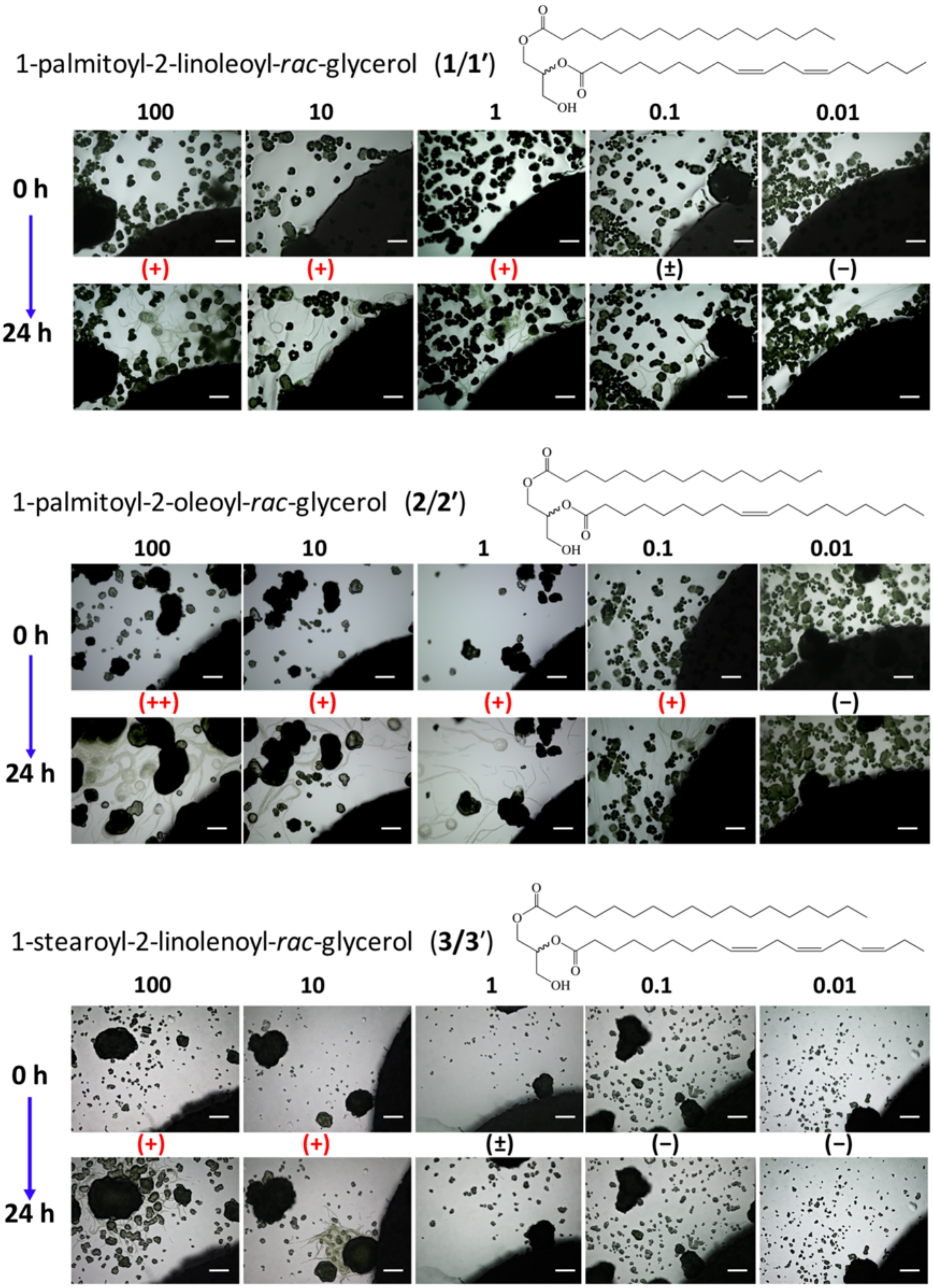

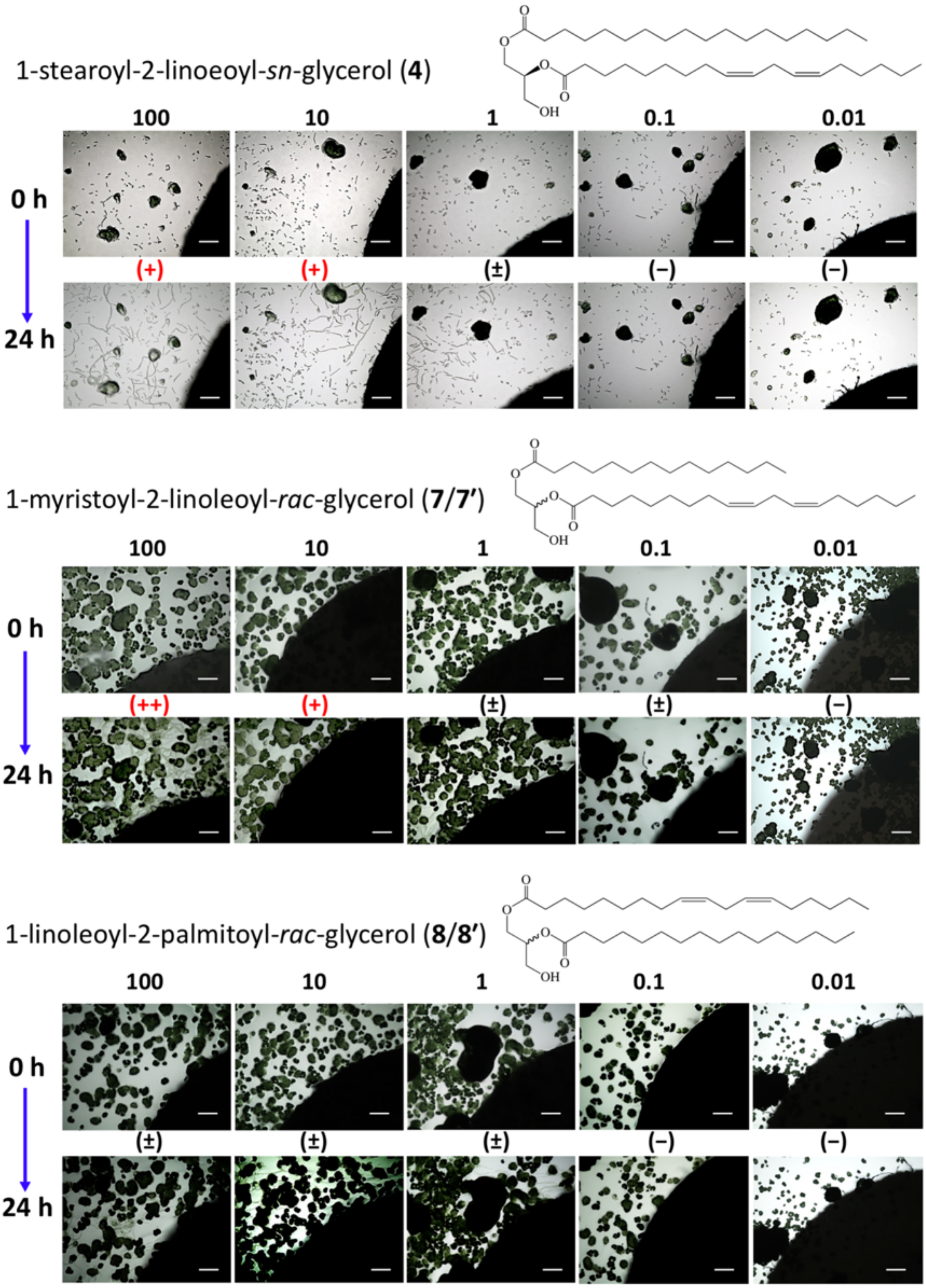

The sandwiched bioassay on BG-11_0-1.2% agar plate. Incubation was performed for 24 h at 23 °C under a 16-h-lighting condition. Synthesized **2**/**2′** showed much more active hormogonia induction than **1**/**1′** at 10-100 nmol/disc. Scale bar represents 500 µm.

Fig. S10. Hormogonium-inducing assay for enantiomeric 1-palmitoylglycerols

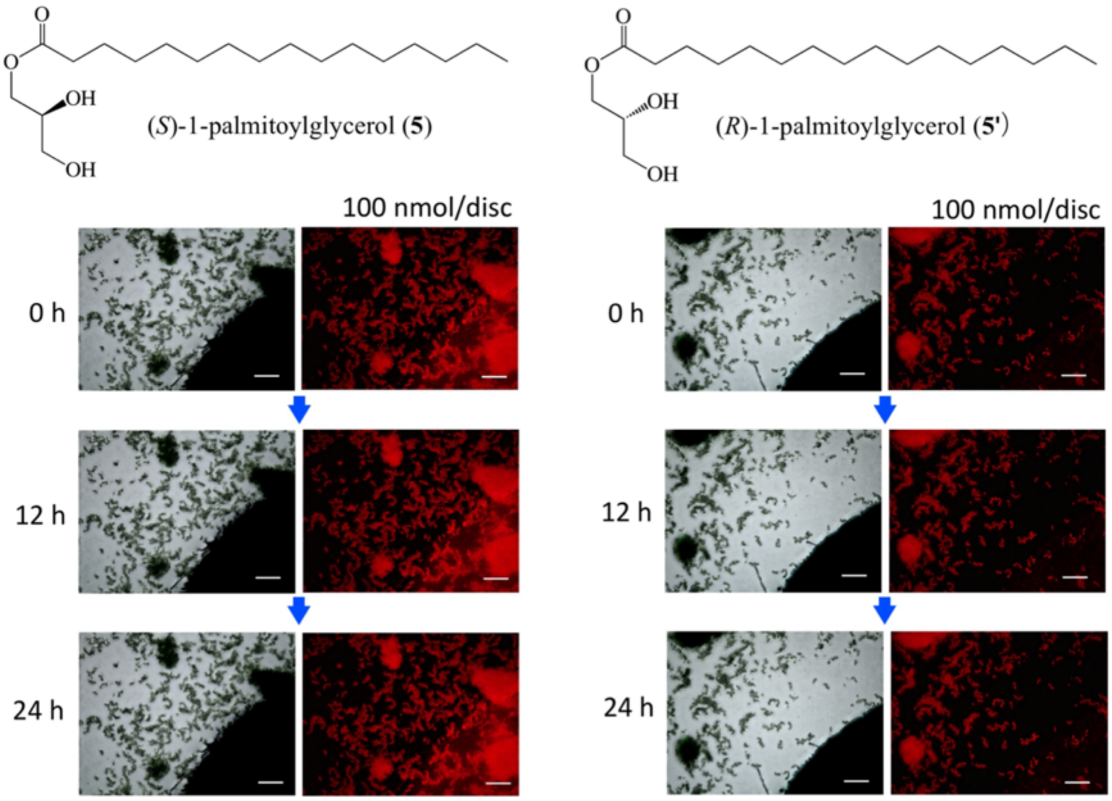

Both enantioselectively synthesised (*S*)-1-palmitoylglycerol (**5**) and (*R*)-1-palmitoylglycerol (**5'**) were tested for the sandwiched bioassay. MAGs were inactive in the bioassay at 100 nmol/disc. Incubation is done for 48h under the bioassay conditions same as described in the text. Scale bar represents 500 μm.

Fig. S11. Hormogonium induction of 1-palmitoyl-2-linoleoyl-*sn*-glycerol obtained from 1-palmitoyl-2-linoleoyl-*sn*-phosphatidylinositol by hydrolysis using phospholipase C

a Enzymatic preparation of **1** from 1-palmitoyl-2-linoleoyl-*sn*-phosphatidylinositol (**10**)

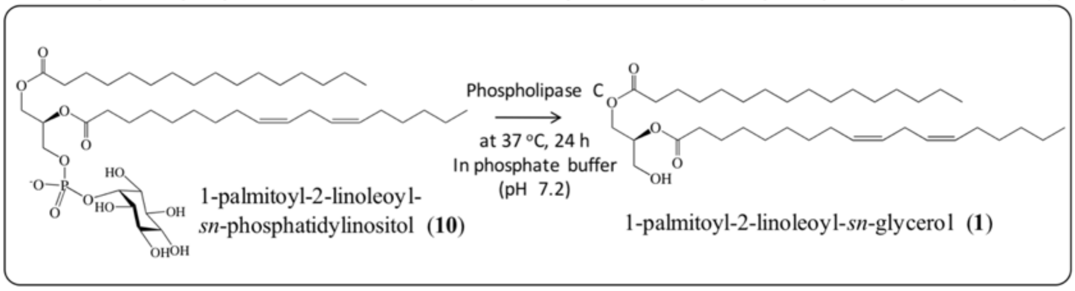

b ^{1}H-NMR spectra of **1** from **10** agreeable with authentic **1/1′**

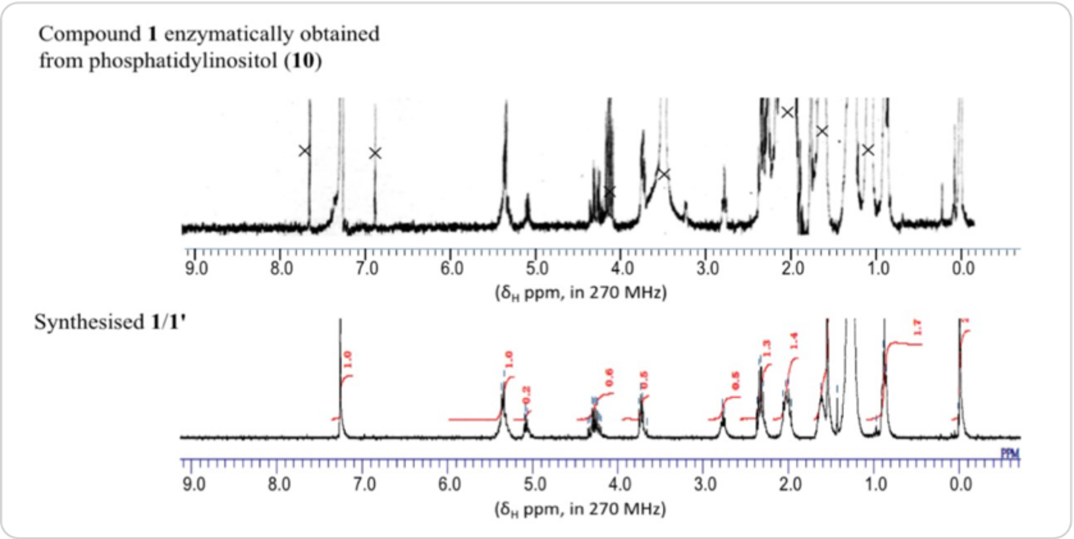

a Enzymatic reaction to obtain 1-palmitoyl-2-linoleoyl-*sn*-glycerol (**1**) from 1-palmitoyl-2-linoleoyl-*sn*-phosphatidylinositol (**10**). Hydrolysis of 1-palmitoyl-2-linoleoyl-*sn*-phosphatidylinositol with phospholipase C (PLC) was done as follows: Enzyme used was PLC from *Bacillus subtilis* parchased from Calbiochem (Darmstadt, Germany). To 1 mL of 50 mM Tris-HCl buffer (pH 7.0) containing 1 mg PLC (60 U), 2.5 mg of 1-palmitoyl-2-linoleoylphosphatidylinositol dissolved in 100 μL dimethyl sulfide was added, and stirred for 8 h at 37 °C. After the reaction, the aqueous solution diluted with 20 mL Milli-Q water was twice extracted with 30 mL EtOAc, and the organic layer was dried over anhydrous Na_2SO_4. The product of the enzyme reaction gave a single spot that reacted positively to vanillin-sulfuric acid reagent and was identical to the 1-palmitoyl-2-linoleoylglycerol that was purified by normal-phase TLC (hexane-EtOAc 4:1). Using ^{1}H NMR, a set of small signals assignable as a DAG were observed. On the basis of a comparison of solvent peaks and olefinic proton signal at δ_H 5.22, its amount was estimated to be 20 μg. This compound (**1**) (yield was less than 5%) was also subjected to the sandwich bioassay to confirm its HIF-like activity. **b** ^{1}H-NMR spectrum of **1** from **10** is identical to authentic**1**/**1′** synthesised in this study.

Fig. S12. Hormogonium-inducing bioassay (impregnation assay) for the enzymatically obtained 1-palmitoyl-2-linoleoyl-*sn*-glycerol from 1-palmitoyl-2-linoleoyl-*sn*-phosphatidylinositol

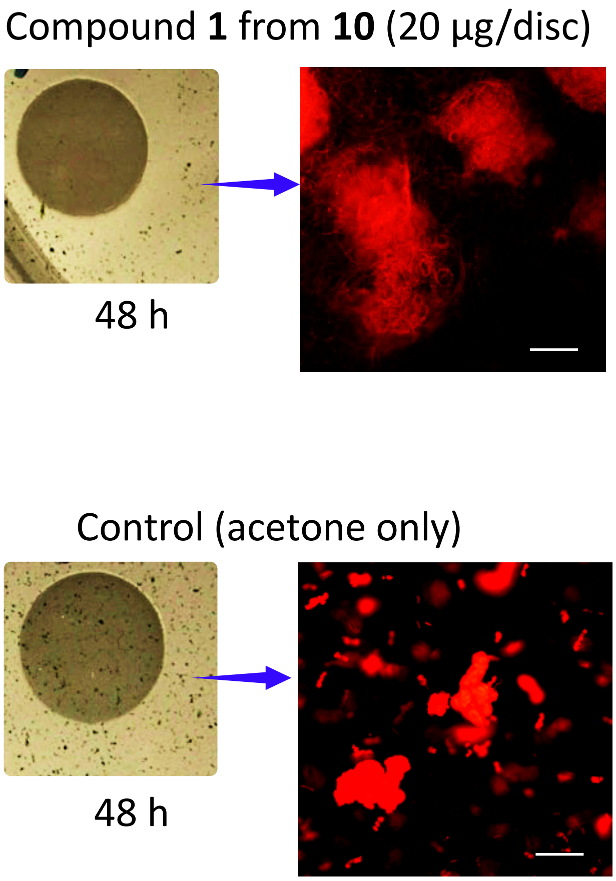

Hormogonium-inducing assay (impregnated bioassay) for enzymatically prepared **1** from **10**. Incubation is for 48 h under the bioassay conditions same as described in the text. Scale bar represents 500 µm.

Fig. S13. Hormogonium-inducing assay for varying amount of anacardic acid C15:1-Δ^{8}

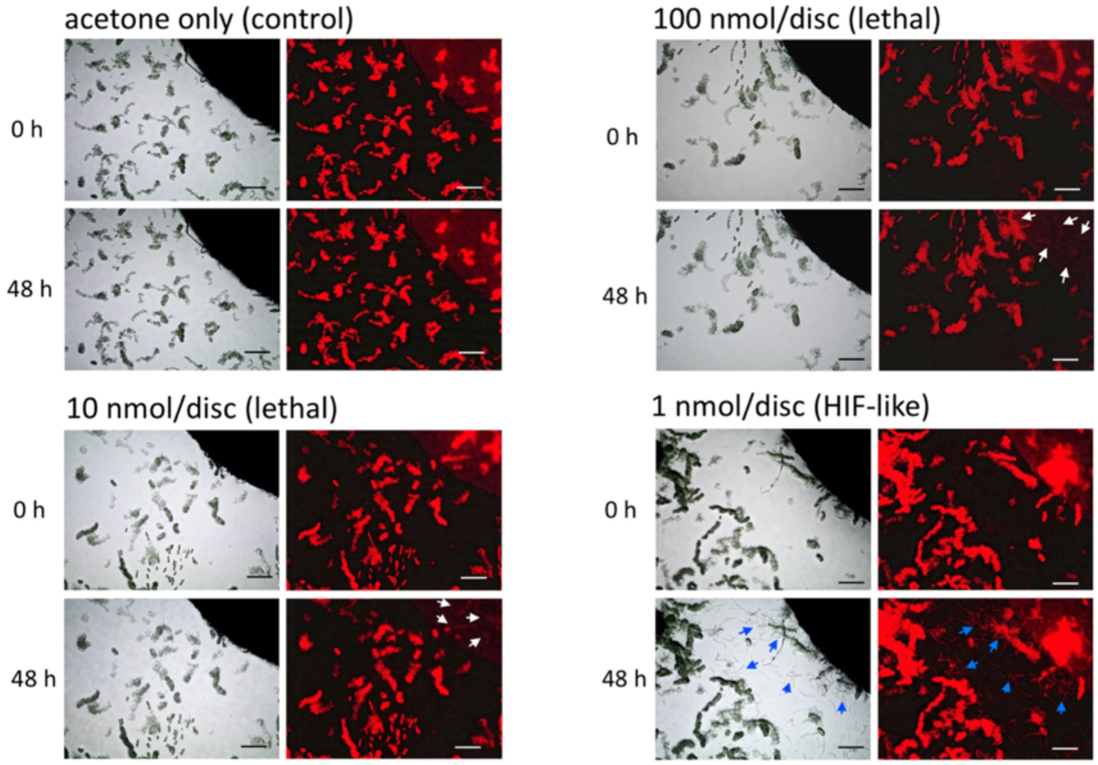

Anacardic acid C15:1-Δ^{8} (**14**) was tested for sandwiched bioassay. Incubation for 48 h under the bioassay conditions is same as described in the text. White arrows in the 10 and 100 nmol/disc of **14** show disappearance of a chlorophyll autofuorescence in cyanobacterial chlorophylls-decomposed area observable only under the paper discs. Blue arrows in the 1 nmol/disc of **14** show induced hormogonia. Note that autofuorescence of chlorohyll a under the paper disc is detectable after 48-h incubation. Scale bars represent 500 µm.
